# Structure of the pecten neuropil pathway and its innervation by bimodal peg afferents in two scorpion species

**DOI:** 10.1371/journal.pone.0243753

**Published:** 2020-12-10

**Authors:** Denise Drozd, Harald Wolf, Torben Stemme

**Affiliations:** Institute of Neurobiology, University of Ulm, Ulm, Germany; Biocenter, Universität Würzburg, GERMANY

## Abstract

The pectines of scorpions are comb-like structures, located ventrally behind the fourth walking legs and consisting of variable numbers of teeth, or pegs, which contain thousands of bimodal peg sensillae. The associated neuropils are situated ventrally in the synganglion, extending between the second and fourth walking leg neuromeres. While the general morphology is consistent among scorpions, taxon-specific differences in pecten and neuropil structure remain elusive but are crucial for a better understanding of chemosensory processing. We analysed two scorpion species (*Mesobuthus eupeus* and *Heterometrus petersii*) regarding their pecten neuropil anatomy and the respective peg afferent innervation with anterograde and lipophilic tracing experiments, combined with immunohistochemistry and confocal laser-scanning microscopy. The pecten neuropils consisted of three subcompartments: a posterior pecten neuropil, an anterior pecten neuropil and a hitherto unknown accessory pecten neuropil. These subregions exhibited taxon-specific variations with regard to compartmentalisation and structure. Most notable were structural differences in the anterior pecten neuropils that ranged from ovoid shape and strong fragmentation in *Heterometrus petersii* to elongated shape with little compartmentalisation in *Mesobuthus eupeus*. Labelling the afferents of distinct pegs revealed a topographic organisation of the bimodal projections along a medio-lateral axis. At the same time, all subregions along the posterior-anterior axis were innervated by a single peg’s afferents. The somatotopic projection pattern of bimodal sensillae appears to be common among arachnids, including scorpions. This includes the structure and organisation of the respective neuropils and the somatotopic projection patterns of chemosensory afferents. Nonetheless, the scorpion pecten pathway exhibits unique features, e.g. glomerular compartmentalisation superimposed on somatotopy, that are assumed to allow high resolution of substrate-borne chemical gradients.

## Introduction

Sensory abilities are of pivotal importance for all animals to navigate through their environment and secure survival of the individual. Foraging for food, finding mates and shelter, avoiding predators and pathogens are all accomplished by the perception and processing of environmental information, including mechano- and chemosensory cues [1–3]. This led to the evolution of a wide variety of unimodal chemosensory and mechanosensory as well as bimodal structures. Chemosensory systems can be divided into olfactory and gustatory pathways, although both senses rest on similar concepts: In both cases chemicals have to access sensory dendrites, first by passing through pores in the body surface, or cuticle in the case of arthropods, and second by binding to transport proteins in the receptor lymph [4–6]. In arthropods, olfactory systems are characterized by a segregation of chemosensory afferents associated with specific receptor types. Each glomerulus in the primary central nervous projection neuropil is typically supplied by just a single type of chemoreceptor–at least in insects [3,7,8]. This principle of olfactory glomeruli as the processing units of chemosensory quality is called chemotopic organisation, and it allows spatial segregation of chemical inputs, thus facilitating extraction of odour qualities [8–10]. Olfactory glomeruli are not only found in arthropod chemosensory systems (Chelicerata: e.g., [11,12], Myriapoda: e.g., [13,14], Crustacea: e.g., [15–18], Hexapoda: e.g., [15,19]) but also in other invertebrate and vertebrate representatives (reviewed by e.g., [3,20,21]). Another modality distributed on the arthropod body in general and on olfactory organs in particular, is mechanosensation. Neuropils innervated by unimodal mechanosensory afferents are typically organised somatotopically (e.g., Chelicerata: [22–25], Myriapoda: [13,14,26], Crustacea: [27–29], Hexapoda: [30–36]). That is, the body surface is mapped on the mechanosensory neuropil, typically warped according to receptor density.

Gustatory/contact-chemosensory sensillae are often bimodal, consisting of several chemosensory and one mechanosensory neuron [36–40]. The afferents of the bimodal chemosensory and mechanosensory neurons project to dedicated neuropils, where inputs from both sensory modalities are organised in a somatotopic fashion (e.g., Chelicerata: [24], Crustacea: [28,29,41], Hexapoda: [36,42–46]).

### Structure and organisation of chemosensory neuropils in chelicerates

In contrast to Mandibulata, chelicerates lack antenna-like structures associated with the second head neuromere, but possess chelicerae instead. Dedicated chemosensory structures–if present–are located on different body regions and are usually distinct and idiosyncratic for the respective taxon [47]. This includes the lyriform and tarsal organs on the tarsi in Araneae [48,49], Haller’s organ on the tarsi in Ixodida [50], the various types of hair sensillae on the first or second pairs of walking legs in Opilliones, Solifugae, Amplypygi and Telyphonida [51–63], the ventrally located chemosensory malleoli on the fourth walking legs in Solifugae [64], or the comb-like pectines in Scorpiones [65,66].

The chemosensory system of mites (Mesostigmata) consists of an olfactory lobe that is located in the first pedal neuromere in the anterior part of the synganglion. It receives input from multiporous sensillae in a dorsal field that is located on the first walking legs, distally on the tarsi [67,68]. The chemosensory axons run along the first pedal nerve and terminate in 14–21 glomeruli [69]. Number and arrangement of glomeruli is largely conserved within a given species. A similar situation is observed in ticks (Ixodida), where olfactory cues from the Haller’s organ are processed in the olfactory lobes [50]. The latter is located antero-ventrally in the first pedal neuropil and consists of grouped small glomerular knots [50,70].

In sun spiders (Solifugae), the antero-ventrally located malleolar neuropil receives input from the fan-like chemosensory malleoli positioned on the ventral sides of the fourth walking legs [12,64,71]. The malleoli afferents proceed along the fourth walking leg nerve. In the prosomal synganglion they form the malleolar tract and project to the malleolar neuropil [12]. The ovoid malleolar neuropils of *Galeodes arabs* consist of up to 13 glomeruli per hemiganglion. The malleolar neuropil is located anteriorly, right ventrally to the pedipalpal neuromere [12].

Bimodal hair sensillae and trichobothria of the spider *Cupiennius salei* are typically located in different positions on the walking legs, with some preference for the distal leg articles, and they send their axons into the prosomal synganglion. Here, both modalities are organised somatotopically, lacking any evidence for glomerular structures [22]. Another distinct projection area is located below the esophagus, between the pedipalpal and the cheliceral neuromeres. These Blumenthal neuropils in spiders are a special ellipsoid neuropil for primary hygro- and thermosensory stimuli but they also integrate mechano- and chemosensory stimuli from the tarsal organs [48]. The neuropils are structured into up to seven columns per hemiganglion that receive input not only from the tarsal organs but also from the hair sensillae of the pedipalps [48].

### The chemosensory pathway in scorpions

**Fig 1:**
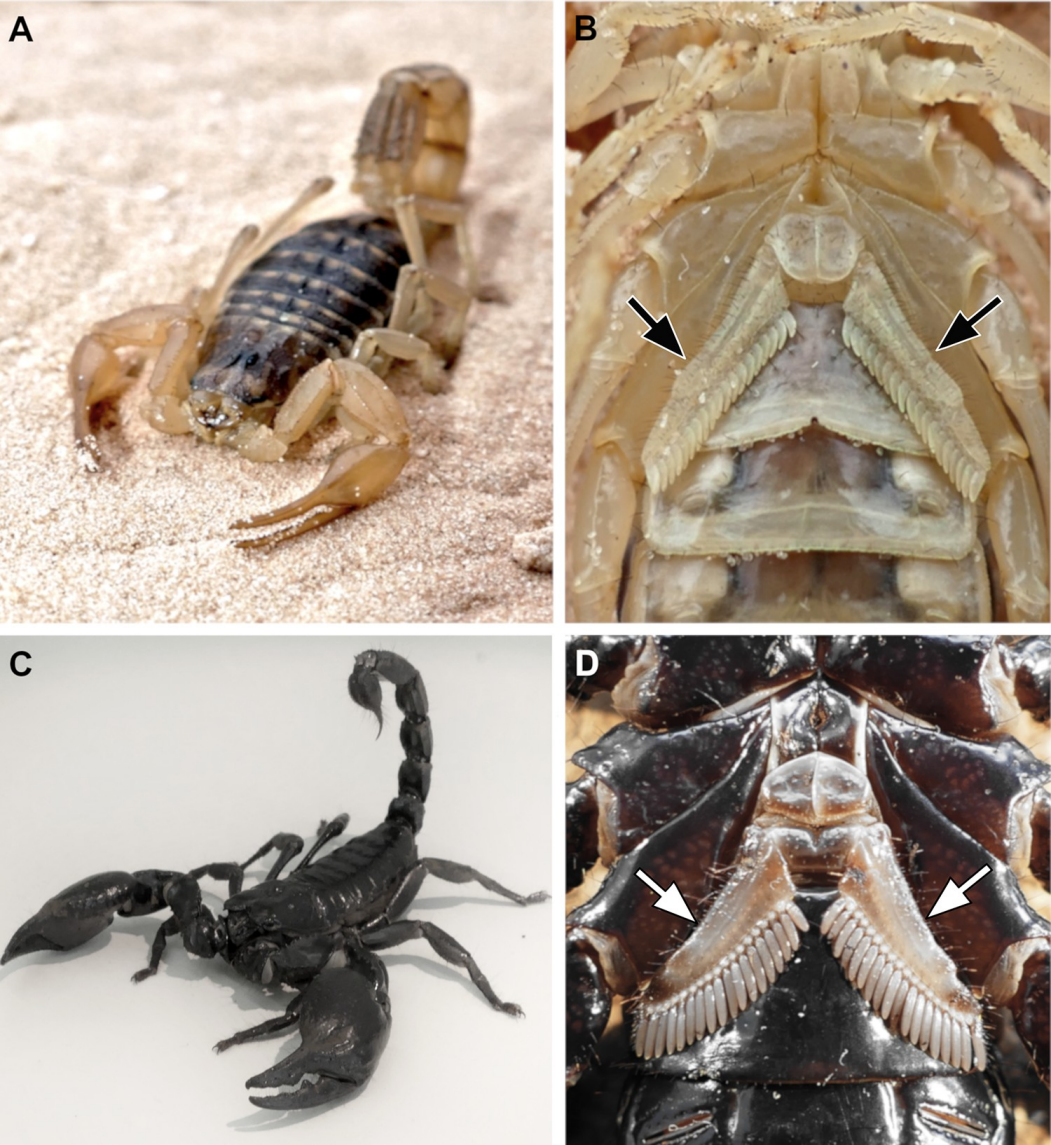
Experimental animals and their specialised sensory appendages, the pectines. (A) *Mesobuthus eupeus* in a natural pose on desert sand. (B) Ventral view of *M*. *eupeus*. Pectines (black arrowheads) are located behind the fourth walking legs on the third mesosomal segment, just posterior the genital operculum. (C) *Heterometrus petersii* in defensive posture. (D) Ventral view of *H*. *petersii*. Pectines (white arrowheads) are light in colour and have wide marginal and medial lamellae.

**Fig 2:**
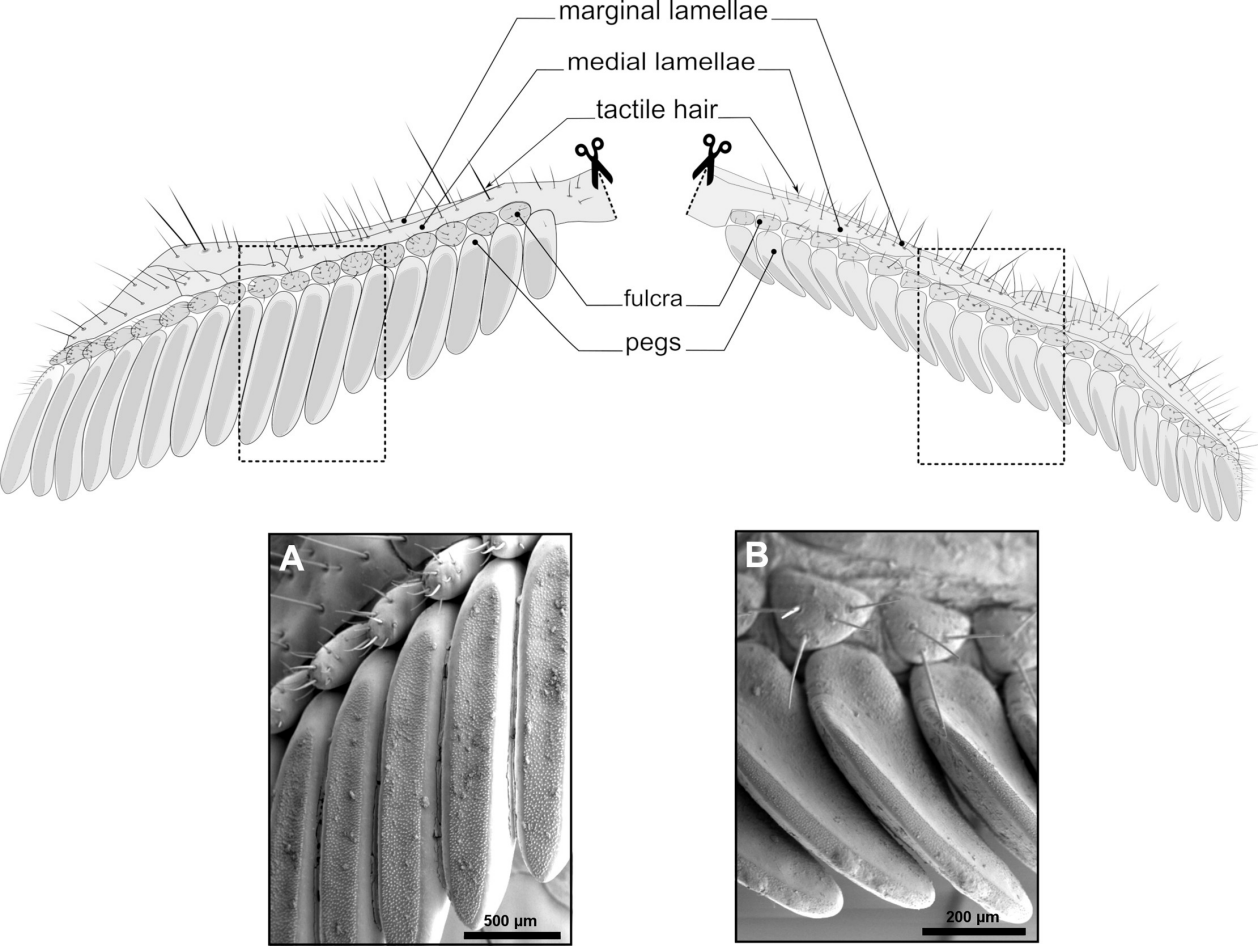
Pectines of *Heterometrus petersii* (left) and *Mesobuthus eupeus* (right). Pectines drawn in natural position, ventral view. Scissors indicate the base of the pecten, where it is connected to the body. Pectines possess marginal lamellae (outer margin of the pectines), medial lamellae (middle section), fulcra (row of more or less dome-shaped mechanosensory pads) and rows of pegs arranged in comb-like fashion. Pegs are studded ventrally with dense fields of peg sensillae (grey areas in diagrams) pointing towards the substrate. All other pecten surfaces are equipped with mechanosensory hair sensillae and trichobothria. (A) and (B) scanning electron microscopic images depicting fulcra and pegs of *Heterometrus petersii* (left) and *Mesobuthus eupeus* (right), respectively.

Scorpions possess pectines located ventrally on the ninth body segment, posterior to the fourth walking legs (Fig 1) [65,72]. The pectines consist of jointed and movable marginal lamellae and medial lamellae, the fulcra and variable numbers of pectinal teeth, or pegs (Fig 2) [11,73,74]. These sensory appendages are oriented towards the substrate and are used to probe and analyse the environment regarding chemo- and mechanosensory stimuli [11,73,75–77]. In general, the external morphology of pectines is rather conserved in scorpions, although number, morphology, and orientation of peg sensillae can vary between species and seem to be related to environmental conditions (Figs 1 and 2) [11,66,75,77–80].

The pectines bear different kinds of receptors to sense both mechano- and chemosensory cues. The lamellae and the fulcra sprout interspersed trichobothria and small hair sensillae [25,81]. The pecten pegs are densely studded with sensillae on their flattened ventral surfaces [11]. This affords a large area for sensillae presentation and resulting high sensitivity, and it puts the sensillae in proximity to the substrate. Peg sensillae are bimodal receptors that include a majority of chemosensory but also mechanosensory neurons [11,75,77]. The sensillae are similar to other arthropod chemo- and mechanoreceptors in their general structure, but have idiosyncratic features [75,82–85]. Peg sensillae extend beyond the cuticle with a stout shaft and contain a distally located slit opening for communication of the sensory dendrites with the environment.

Pectines are suggested to function as substrate/contact chemosensory organs, involved in mate localisation and, with lesser evidence, in prey trailing and localisation [76,86–90]. It has further been proposed that scorpions use path integration without the necessity of visual input during homing [91]. The pectines seem to be involved in navigation in general and homing in particular, by using scene familiarity detected by the peg sensillae [92,93].

The afferent neurons from the hair and peg sensillae convey different environmental cues to the pecten neuropil via the pecten nerve: Hair sensillae receive tactile input, whereas peg sensillae receive both chemical and mechanical stimuli. The pecten nerve enters the synganglion from the ventro-posterior side and ends in the pecten neuropil. The pecten neuropil is subdivided into two major neuropil parts, the posterior pecten neuropil, located behind the fourth walking leg neuromere, and the anterior pecten neuropil on the level of the third walking leg neuromere. The latter is connected to the posterior pecten neuropil by a longitudinal tract [11,94]. The afferents of the bimodal sensory sensillae of the pectines project to the pecten neuropil in a somatotopic fashion, although it remains unknown whether or not chemo- and mechanosensory afferents are superimposed within the pecten neuropil or project to different neuropil parts [11,94]. The size of the pectines, the number of pegs and of peg sensillae, and the morphology of the pecten neuropils depend on species and sex [11,75].

The present comparative study focuses on the central nervous projections of afferent fibres of single pegs in the pecten neuropils of two scorpion species. The animals examined in this study are the desert scorpion *Mesobuthus eupeus* (Koch, 1939) (Buthidae), a 5–6 cm long yellow-brown scorpion that lives in hot and arid habitats between eastern Turkey and northern China (Fig 1A and 1B). The pectines of *Mesobuthus eupeus* are 3–4 mm in length, with up to 24 pegs per pecten. The pegs of *M*. *eupeus* are small and curved, with a sensory field of peg sensillae oriented in the direction of the substrate (Fig 2). *Heterometrus petersii* (Thorell, 1876) (Scorpionidae) is one of the largest extant scorpion species with a body length of up to 11 cm (Fig 1C and 1D). This dark-coloured species lives in Vietnam, Laos and Cambodia, where it inhabits dense humid jungle forests and digs its burrow into the soil. Its pectines bear up to 16 pegs per pecten and are approx. 6 mm in length (Fig 2). The two scorpion species illustrate different physiological characteristics related to their respective biological habitats and are phylogenetically rather distantly related. Differences in the morphology of the pectines and their respective neuropils may thus provide a better understanding of chemosensory processing in scorpions in a neuroanatomical context. We employed neuroanatomical techniques to study peg afferent innervation and neuropil organisation, and to reveal the sorting of chemosensory inputs according to spatial qualities.

## Material and methods

### Experimental animals

*Mesobuthus eupeus* and *Heterometrus petersii* were obtained from The Pet Factory (www.thepetfactory.de). Adult animals were kept separate in plastic boxes (13.5 x 14.5 x 8 cm for *M*. *eupeus* and 57 x 39 x 28 cm for *H*. *petersii*) under artificial light conditions (14 h light /10 h dark) and at temperatures around 27°C. The boxes were filled with 3 cm sand for *M*. *eupeus* and 10 cm soil for *H*. *petersii* as substrates. Stones and bricks served as shelters. Small shallow water dishes were provided, and three times a week the water was renewed. Feeding occurred every two weeks with small (larval stages 2 and 3) house crickets (*Acheta domesticus*) for *M*. *eupeus* and subadult (larval stage 5) locusts (*Schistocerca gregaria*) for *H*. *petersii*, purchased from Fressnapf (Ulm, Germany). For the experiments, 40 animals of *M*. *eupeus* and 25 of *H*. *petersii* were analysed.

### Anterograde tracing of afferent fibres with Neurobiotin

Specimens of *M*. *eupeus* were anaesthetised with 99.7 Vol.% CO_2_ and cooled down to 4°C in the refrigerator for 45 min (n = 30). *H*. *petersii* exhibited a drastic reaction to this treatment which resulted in the death of several individuals. Therefore, we only cooled *H*. *petersii* in the refrigerator for 30 min and did not use CO_2_ (n = 25).

**Fig 3:**
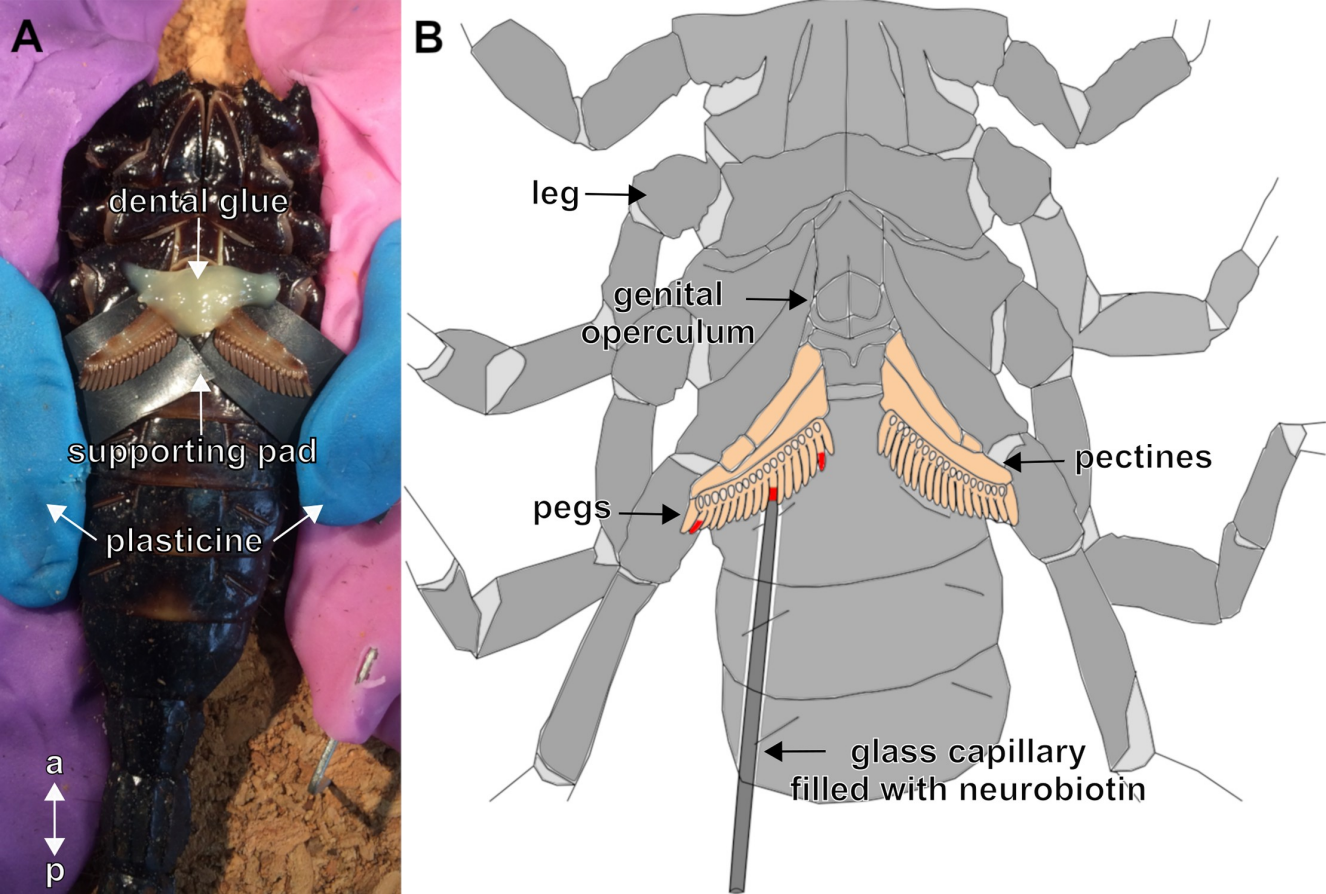
Experimental set-up for anterograde tracing of peg afferents. (A) Photo of a cold-anaesthetised *Heterometrus petersii* fixed to a cork board, ventral side up. Pectines were cleaned, stabilised with plasticine, fixed into position with dental glue and supported with a plastic film. (B) Sketch of the anterograde tracing procedure using Neurobiotin. Pectines are highlighted in beige, examples of incisions at individual pegs in different positions marked in red. Glass capillaries filled with Neurobiotin were put over the incision. Abbreviations: a: anterior; p: posterior.

All following preparations were done under a binocular dissection microscope (stereomicroscope Stemi 508, Zeiss, Germany) for better visualisation. The cooled animals were immobilised dorsal side up onto cork plates with plasticine, rendering the ventral appendages accessible. The pecten bases were immobilised with dental glue (3M^™^ Protemp™ II, Germany), stabilised by a supporting pad (Fig 3A), and cleaned with demineralised water (demin. H_2_O). To label all sensory fibres, the pecten was cut approx. 0.5 cm distal to its base. The wound was immediately filled with demin. H_2_O to cause axon swelling, in case any axons had been squeezed in the process. In the meantime, a small well of petrolatum was created around the incision site, and after 5 minutes, the water was removed with a small piece of lab tissue. 10 μl of 5% Neurobiotin Tracer (Neurobiotin™, SP-1120, Vector Laboratories, Canada) dissolved in demin. H_2_O was filled into the well, covering the incision site, and was sealed with petrolatum. The animals were stored at 4°C for 48 h.

To analyse innervation patterns of afferent fibres originating from individual pegs, we used glass capillaries (Harvard Apparatus LTD, USA) of the type GC100TF– 10 (1.0 mm outer diameter, 0.78 mm inner diameter). After pulling the capillaries (Sutter Instrument CO., Model P-97, USA), the tip of the glass capillary was cut off to fit the pectinal tooth and filled with 5% Neurobiotin tracer dissolved in demin. H_2_O. Because the pegs of *H*. *petersii* were considerably larger than those of *M*. *eupeus*, the glass capillaries used for *H*. *petersii* were not usually pulled into a tip. One pectinal tooth on each pecten of an individual was cut apically with sharp small scissors (Fine Science Tools, Germany) and rinsed with demin. H_2_O for 5 min. For the different experiments, individual pegs at different distances from the body were severed. Numbering of the pegs for identification was from the base to the distal end of the pecten. After rinsing, the water was removed with a small piece of tissue and the filled glass capillary was put over the previously severed pectinal tooth. Fig 3B depicts a sketch of the experimental setup. To prevent desiccation and the animal from moving, dental glue was applied to the top of the pecten as well as the back of the capillary.

*M*. *eupeus* were stored in moist chambers in the refrigerator at 4°C for 48 h, and *H*. *petersii* were stored in a cool room at 15°C for 48 h. This was because of the sensitive reaction of *H*. *petersii* when exposed to temperatures below 15°C for a longer period of time.

### Dissection and fixation

Glass capillary, plasticine and dental glue, or in case of the anterograde tracing the whole pecten nerve and the petrolatum were removed. The ventral body plate with the attached synganglion was carefully removed with the help of two forceps and fine scissors and transferred to a shallow glass bowl filled with phosphate-buffered saline (PBS, 10 mM sodium phosphate, 150 mM sodium chloride, pH 7.4) (chemicals obtained from Merck, Darmstadt, Germany). The synganglion was separated from the surrounding tissue and rinsed in fresh PBS. The nervous tissue was fixed by incubation with 4% paraformaldehyde (PFA) (Sigma-Aldrich, St. Louis, MO) in PBS for 24 h at 4°C, followed by three washing steps in PBS, 15 min each. 7% low-melting point agarose (Roth) was heated to approx. 40°C. The synganglia were transferred to black scale pans, carefully dried with filter paper, and coated with Poly-D-Lysine (1mg/ml in demin. H_2_O, Specialty Media, Merck, Darmstadt, Germany). The coated synganglia were embedded in the prepared agarose. After the agarose had set, the blocks were trimmed with a razor blade and sectioned on a Leica VT1000 S Vibratome (Leica Biosystems, Wetzlar, Germany) to 75 μm thick horizontal, frontal or sagittal sections. Slices were collected in a 12 well cell culture plate (Costar®, Corning Incorporated, USA) filled with ice-cold PBS.

### Immunohistochemistry

Sections were permeabilised with 0.3% Saponin (Fluka BioChemika, USA) in PBS containing 0.3% Triton X-100 (PBS-T 0.3%; Sigma-Aldrich) for 1 h at room temperature (RT). Subsequently, the tissue was washed in PBS-T 0.3% or PBS, respectively, for 30 min and blocked for 3 hours at room temperature (RT) with a blocking solution, consisting of 5% normal goat serum (Vector Laboratories, USA) in PBS-T 0.3%. To visualise synapsin-rich regions or axonal tracts, the primary antibodies mouse-anti-SYNORF1 (3C11, Developmental Studies Hybridoma Bank, University of Iowa, USA; final concentration 1:70) or rat-anti-tyrosinated tubulin (ab6160, Abcam, Cambridge, UK; final concentration 1:2000), respectively, were applied in blocking solution to the freely floating sections for 3 h at RT or overnight at 4°C. After three washing steps with PBS-T 0.3%, 15 min each, sections were incubated in the secondary antibodies goat-anti-mouse Alexa Fluor^®^ 488-conjugated (Invitrogen, Thermo Fisher Scientific Life Technologies, Darmstadt, Germany), goat-anti-mouse Cy3-conjugated (Invitrogen, Thermo Fisher Scientific Life Technologies), or goat-anti-rat Alexa Fluor 555-conjugated (Invitrogen, Thermo Fisher Scientific Life Technologies), respectively, each diluted 1:250 in blocking solution. Streptavidin Cy3-conjugated (S6402-1ml, Sigma-Aldrich) was added 1:250 for visualisation of the Neurobiotin tracer, and 4’,6-diamidine-2-phenylindole-dihydrochloride (DAPI; 1 μg/ml) (D9542 Sigma-Aldrich) to label cell nuclei. The tissue was then incubated over night at 4°C. Two washing steps with PBS-T 0.3% and a final step with PBS followed, 15 min each. The sections were mounted on microscope slides (Thermo Scientific™ SuperFrost Plus™, Thermo Fisher, Schwerte, Germany) and covered with Mowiol® 4–88 (Merck) as mounting medium agent.

### Lipophilic dye labelling in fixed tissues

To examine the innervation pattern of afferents of closely located pegs simultaneously, we applied labelling using two lipophilic tracers, allowing discrimination due to their specific emission wavelengths. Therefore, we used DiI (1,1′-Dioctadecyl-3,3,3′,3′-tetramethylindocarbocyanine perchlorate, Sigma-Aldrich, Munich, Germany) and DiA (4-(4-(Dihexadecylamino)styryl)-N-methylpyridinium iodide, ATT Bioquest, Biomol, Hamburg, Germany) in fixed tissue of *Mesobuthus eupeus* (n = 10).We removed the scorpion’s ventral plate, with pectines intact and central nervous system attached, and fixed the specimens for 24 h in 4% PFA in PBS at 4°C. Afterwards, the tissue was washed three times in PBS, 15 min each, with gentle agitation on a shaker. The ventral plate was pinned, pectines pointing up, to the bottom of a petri dish covered with Sylgard 184 (Dow Corning, Farnell, Aschheim, Germany) and filled with PBS. A small amount of the lipophilic tracer was dissolved in methanol (Merck) and vortexed. A pulled glass capillary with broken tip was coated with the tracer-methanol mix, and the methanol left to evaporate. Meanwhile, the tips of differently located pegs were cut off with small scissors and afterwards, a coated glass capillary was inserted into the lacerated peg to place the fluorescent paste inside the peg. This was repeated until the peg was filled with a considerable amount of lipophilic tracer. The ventral plates were put into 50 ml falcon tubes (Cellstar®, Greiner Bio-One, Frickenhausen, Germany) filled with PBS containing 0.3% sodium azide (NaN_3_, Carl Roth) and stored in a heat cabinet at 30°C for approx. 3 to 5 weeks. The PBS was changed weekly and the pectines were examined by means of a dissection microscope to check whether the dye had reached the nervous system. Finally, the peripheral nerves were cut and the central nervous system was separated from surrounding tissue with fine forceps and minuten scissors. After vibratome sectioning (see above), slices were treated for immunolabelling against synapsin. Immunolabelling followed the protocol above with some modifications: Permeabilisation with saponin and Triton-X100 was omitted to maintain the stability of membranes and to avoid fading of the lipophilic tracer labelling. Thus, 1 mg/ml digitonin (D141, Sigma-Aldrich) was added as a detergent to the incubation media [95]. Since digitonin is not soluble at RT, the solution was heated to 90°C, cooled down and applied onto the sections.

### Antibody specificity

To identify neuropilar structures and facilitate orientation within the nervous system of the scorpions, we used the monoclonal mouse anti-*Drosophila* synapsin antibody (“SYNORF1”, 3C11, Developmental Studies Hybridoma Bank, University of Iowa, USA; final concentration 1:70). This antibody was raised against a *Drosophila* Glutathione S-Transferase (GST)-synapsin fusion protein. The antibody reacts with a highly conserved epitope, as it labels neuropilar structures in many euarthropod taxa [13,16,96–99] including arachnids [100,101]. Recently, this antibody has been applied as structural marker in the central nervous system of the scorpion *Euscorpius italicus*, resulting in the labelling of all neuropil structures, e.g. the arcuate body and pecten neuropils [101].

The rat monoclonal tubulin antibody [YL1/2] (ab6160, Abcam, Cambridge, UK; final concentration 1:2000) was used to label axonal fibre tracts, due to its ability to recognise tyrosinated alpha tubulin and its binding to other targets that contain a negatively charged carboxy terminus such as recA and oxidised actin (see manufacturer datasheet). Tubulin is the major constituent of microtubules and represents a heterodimer of α-tubulin and β-tubulin [102]. The antibody reacts with tyrosinated tubulin from mammals such as mouse and african green monkey (*Chlorocebus sabaeus*), amphibians (*Xenopus laevis*), nematodes (*Caenorhabditis elegans*), insects (*Drosophila melanogaster*) and other organisms, such as yeast (*Saccharomyces cerevisiae*) (manufacturer datasheet), indicating that the antigen that this antibody recognises is highly conserved across a broad range of species.

### Image acquisition and analysis

Z-stacks of sections were acquired with a confocal laser-scanning microscope TCS SP5 II (Leica Biosystems) with the respective Leica LAS AF software. To examine these neuroanatomical data, the respective image stacks were loaded into ImageJ1.51n (Rasband, W.S., ImageJ, U.S. National Institutes of Health, Bethesda, MD, http://rsb.info.nih.gov/ij/) and edited. Editing consisted of only global adjustments of brightness and contrast as well as changes of colors. Relevant image stacks can be provided upon request. Figure panels were arranged in Gimp 2.8 (www.gimp.org). Sketches for illustrating the findings were drawn in INKSCAPE 0.92.4 (www.inkscape.org/de).

## Results

### General remarks on the central nervous system of the investigated species

The nervous systems of the desert scorpion *M*. *eupeus* and the tropical scorpion *H*. *petersii* do not differ in their general anatomy, nor are there differences with respect to other scorpion species. Only the size of the nervous system differs in relation to the body size. The antero-dorsal part consists of the protocerebrum housing the visual neuropils, the mushroom bodies and the arcuate body. The deutocerebrum supplies the chelicerae, and the tritocerebrum represents the pedipalpal neuromere. The esophagus passes from the ventral to the dorsal side along the midline in between the deuto- and tritocerebrum. The tritocerebral neuromere is thus anatomically part of the ventral nerve cord. The anterior nerve cord ganglia are fused into a synganglion that includes the proto-, deuto- and tritocerebral ganglia, the four walking leg, and the mesosomal genital and pecten neuromeres.

### General morphology of the pecten neuropils in scorpions

**Fig 4:**
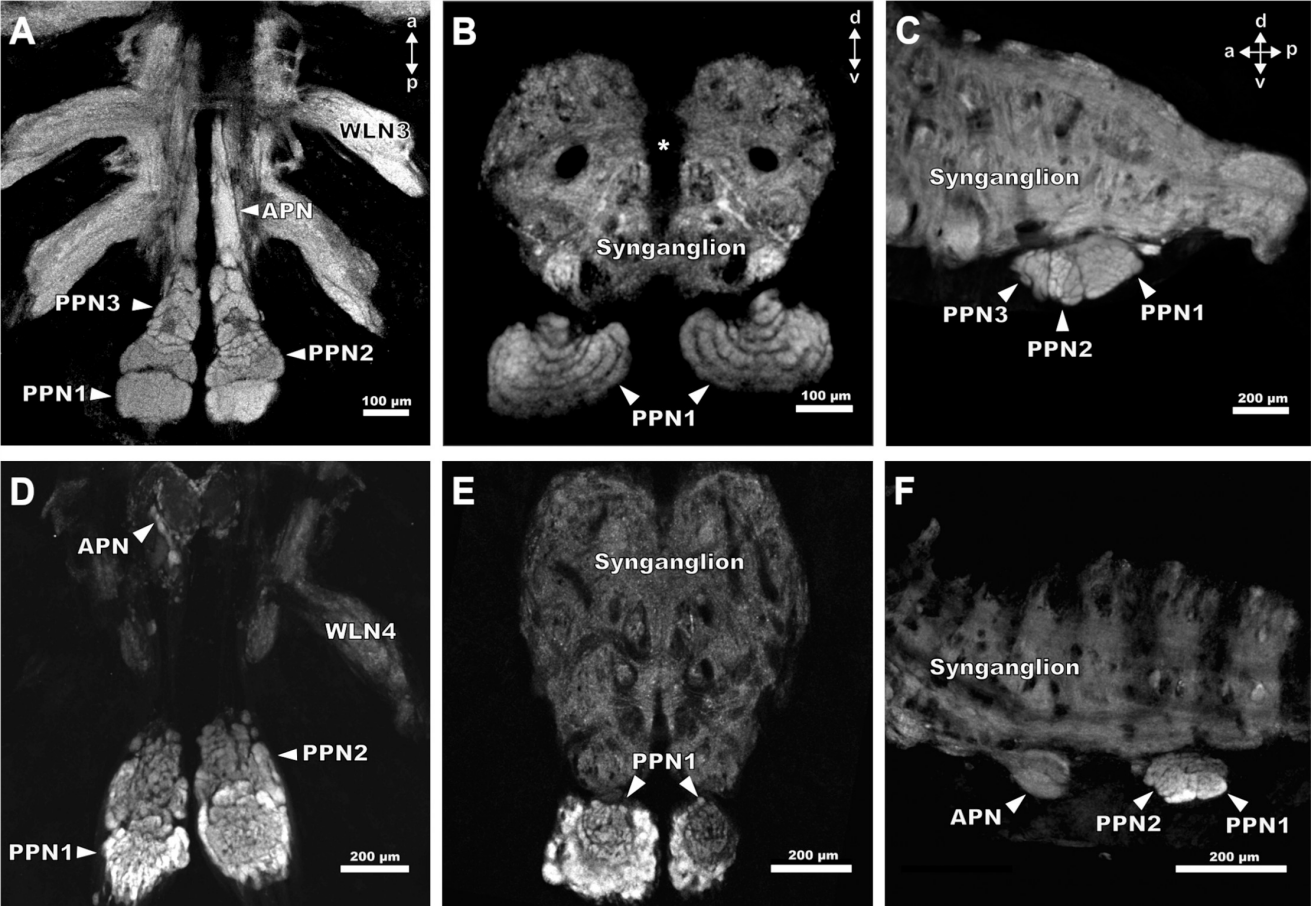
Position and morphology of the pecten neuropils in *Mesobuthus eupeus* (A-C) and *Heterometrus petersii* (D-F). Maximum projections of cLSM stacks, showing neuropilar regions in grey (synapsin-immunoreactivity). (A) Horizontal section of the posterior part of the synganglion of *M*. *eupeus*. The associated neuropils of the pecten extend from the most posterior region anteriorly to the level of the third walking leg neuromere (WLN3). The pecten neuropil is divided into the anterior pecten neuropil (APN) and the posterior pecten neuropils 1–3 (PPN 1–3). (B) Frontal section of the PPN and synganglion located just dorsally. The PPN1 appears to be layered, with “onion peel”-like arches that are smaller in dorsal direction and wider in ventral direction. The area between the hemiganglia is slightly torn (asterisk). (C) Sagittal section of the synganglion of *M*. *eupeus*. PPN protrudes ventrally from the synganglion. (D) Horizontal view of PPN of *H*. *petersii*. Immunolabelling appears stronger in PPN than in the remaining synganglion. PPN1 has a stronger synapsin-immunoreactivity in the outer cortex, compared to the central region. These subcompartments are not as differentiated as in *M*. *eupeus*. APN is ellipsoid and heterogeneously structured, exhibiting a weaker immunoreactivity of synapsin than PPN. (E) Frontal section of the synganglion of *H*. *petersii*. The outer cortex of PPN1 appears to be more nodular than the inner layers with stronger synapsin immunoreactivity. (F) Sagittal section of the synganglion of *H*. *petersii*. APN is very prominent and positioned ventrally at the level of the WLN3. PPN protrudes ventrally from the synganglion. Abbreviations: a: anterior; APN: anterior pecten neuropil; d: dorsal; p: posterior; PPN1-3: posterior pecten neuropil 1–3; v: ventral; WLN3-4: walking leg neuromere 3–4.

**Fig 5:**
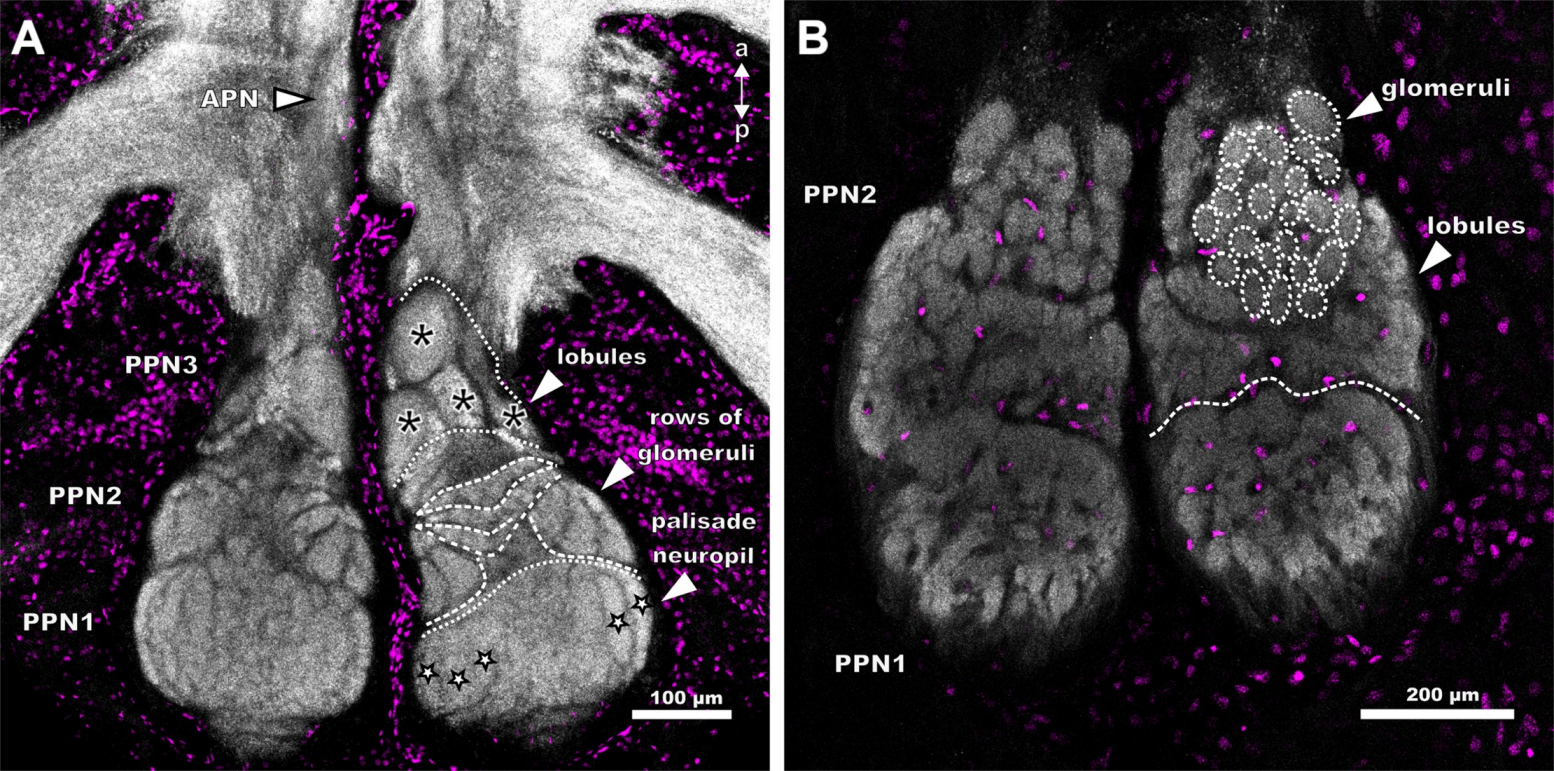
Detailed view of the posterior pecten neuropils of *Mesobuthus eupeus* (A) and *Heterometrus petersii* (B). Maximum projections of horizontal cLSM stacks. Neuropilar tissue in grey and nuclear labelling in magenta. (A) The three major posterior pecten neuropils (PPN1-3) of *M*. *eupeus* consist of numerous subcompartments: PPN1 consists of palisade-shaped neuropilar regions (outlined stars). Two large lobules (white dashed lines) are located at the lateral and medial side of the PPN2 with wedge-shaped neuropil subcompartments. An area of arcuate rows (white dashed lines) consisting of smaller glomeruli, is positioned between the lobules. PPN3 is built up of four lobules (asterisks). The anterior pecten neuropil (APN) extends anteriorly. (B) PPN of *H*. *petersii* is interspersed with somata and divided in the heterogeneously structured PPN1 and 2 (white dashed line). PPN2 consists of two lobules, located at the lateral and medial sides of the neuropil and numerous glomeruli (white dotted lines). Abbreviations: a: anterior; APN: anterior pecten neuropil; p: posterior; PPN1-3: posterior pecten neuropil 1–3; WLN4: walking leg neuromere 4.

The neuropils associated with the pectines extend from the posterior end of the synganglion to the third walking leg neuromere. Sensory information is conveyed via the pecten nerve that enters the pecten neuropil postero-ventrally. These pecten neuropils are subdivided into two main regions: the posterior pecten neuropils 1–3 (PPN1-3) and the anterior pecten neuropil (APN) (Fig 4). PPN1 and PPN2 form an ellipsoid neuropil in the posterior third of the synganglion, postero-ventrally to the fourth walking leg neuropils (Fig 4A and 4D). PPN3 is located near the ganglion midline, extends anteriorly and connects the posterior neuropils to the APN. The latter terminates at the level of the third walking leg neuromere (Fig 4A). Each neuropilar subregion exhibits a distinct morphology and anatomy, thus making it distinguishable from the other neuropils in the nervous system (e.g. the walking leg ganglia, Fig 4A). The PPN1 exhibits a lamellar structure, which is more or less distinct, depending on species (Fig 5A and 5B). PPN3 extends anteriorly and contains lobules, whereas PPN2 consists of an anterior glomerular region and a lateral as well as medial lobular region (Fig 5A). We did not observe any differences regarding neuropil structure related to sex, although the pecten neuropils seem to be larger in specimens with larger number of pegs.

### Structure of the pecten neuropils in *Mesobuthus eupeus*

The pecten neuropil of *M*. *eupeus* is characterised by a clear demarcation of the individual neuropil parts (PPN1-3, APN) and each subregion exhibits a specific and unique compartmentalisation (Figs 4A and 5A).

**Fig 6:**
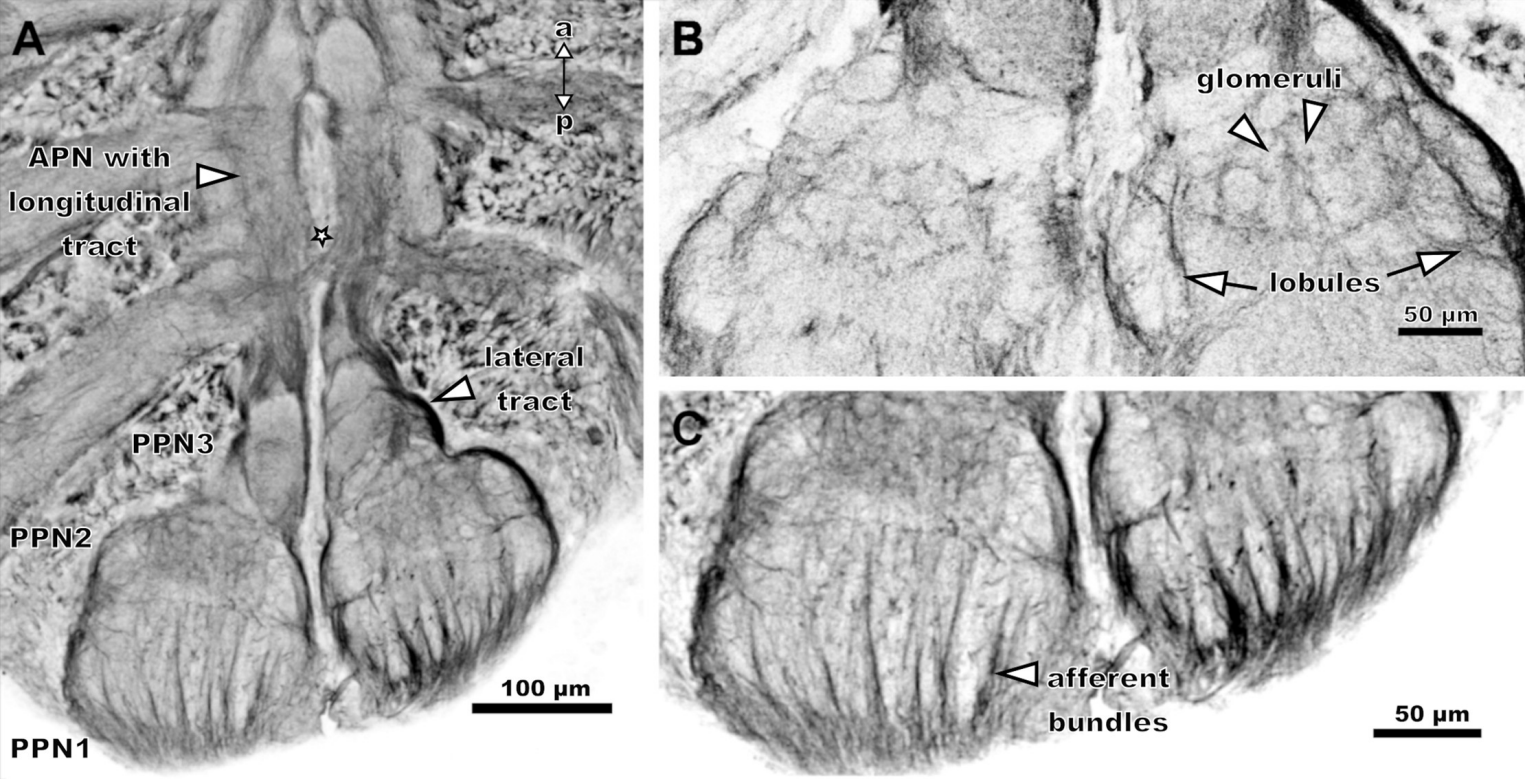
Axonal pathways of the pectines in *Mesobuthus eupeus*. **. Maximum projections of horizontal cLSM stacks.** Tubulin-immunoreactivity: grey/black. (A) A major tract runs along the posterior pecten neuropil (PPN) laterally (outlined arrowhead). The anterior pecten neuropil (APN) includes a longitudinal tract (outlined arrowhead). Hemiganglia are connected by commissure (outlined star). (B) Higher magnification of PPN2. The glomeruli (outlined arrowheads) and bilateral lobules (outlined arrows) are demarcated by axonal fibres. (C) Higher magnification of PPN1. Afferent bundles are located at the posterior end and are organised in parallel fashion (outlined arrowhead). Abbreviations: a: anterior; APN: anterior pecten neuropil; p: posterior.

The PPN1 is located more dorsally than the other parts of the pecten neuropil (Fig 4C) and organised in a laminar fashion. The single lamellae do not appear to be strictly parallel, but rather crescent-shaped (Fig 5A). In frontal sections, this region appears structured in an onion-peel fashion, with six arcuate layers (Fig 4B). The lamellae are surrounded by afferent bundles running in parallel and entering the pecten neuropil via the pecten nerve from the postero-ventral side (Fig 6A and 6C). Further an afferent tract proceeds anteriorly along the lateral and medial margins of the pecten neuropil, innervating all neuropil regions along their paths (Fig 6).

The PPN2 includes two lobules that consist of wedge-like subcompartments (Figs 5A and 6B). The area between and anterior to the lobules contains three arcuate rows of glomeruli-like neuropilar structures, which terminate anteriorly in the PPN3 (Figs 4A, 5A and 6B). Axonal fibres delimit the larger lobules and the smaller glomeruli (Fig 6B).

The cylindrical PPN3 is located anteriorly to PPN2 and consists of lobe-shaped neuropils, which are organised in a pinecone-like fashion (Figs 5A and 6A). A lateral tract partially innervates the PPN3 and proceeds anteriorly. They converge along the ganglion midline within the APN (Figs 4A, 5A and 6A).

The APN is situated anteriorly to PPN3, near the ganglion midline and ascends to the level of the third walking leg neuromere (Figs 4A and 6A). It consists of a homogenously structured neuropil (Figs 4A and 6A). At the level of the fourth walking leg, the two hemiganglia are connected by a commissure (Fig 6A). It could not be determined if this commissure is associated with the pecten neuropils.

### Structure of the pecten neuropils in *Heterometrus petersii*

The pecten neuropils of *H*. *petersii* have a dumbbell shape, with a prominent ellipsoid PPN and an enlarged APN with bulbous subcompartments (Fig 4D). The respective subregions (PPN1,2) exhibit a more intricate compartmentalisation and an overall more intertwined neuropilar anatomy compared to *M*. *eupeus* (compare Figs 4A, 4D, 5A and 5B). Furthermore, the inner regions of the PPN are interspersed with a rather high number of somata in *H*. *petersii* (Fig 5B).

The PPN1 has a disc-like appearance and higher concentrations of synaptic terminals, which results in stronger synapsin-immunoreactivity compared to the other neuropil areas (Fig 4D–4F). The lamellar structure of PPN1 is more intertwined and not arranged in a parallel fashion like in *M*. *eupeus* (compare Fig 5A and 5B). In frontal sections this area appears to be arranged in concentric layers, with a stronger synapsin-immunoreactivity in the outer cortex (Fig 4E).

The PPN2 is subdivided into two lobules, which do not consist of wedge-shaped but rather laminar neuropil regions (compare Fig 5A and 5B). Three to four rows of glomeruli are situated anterior to the lateral lobules. Anterior to PPN2, there is an area of larger glomerular structures (sometimes more lobular). This makes it difficult to determine whether this region can be defined as a separate PPN3 or if it belongs to PPN2. The borders are not as distinct as in *M*. *eupeus* and the region does not exhibit distinct lobular structures (Fig 5B).

The main difference between the two species is in the anatomy of APN. The relative size of this neuropil appears to be significantly larger in *H*. *petersii*. Further, several spheroid substructures can be observed in the APN (Fig 4D).

### Projection areas of the pecten afferents in the pecten neuropil

**Fig 7:**
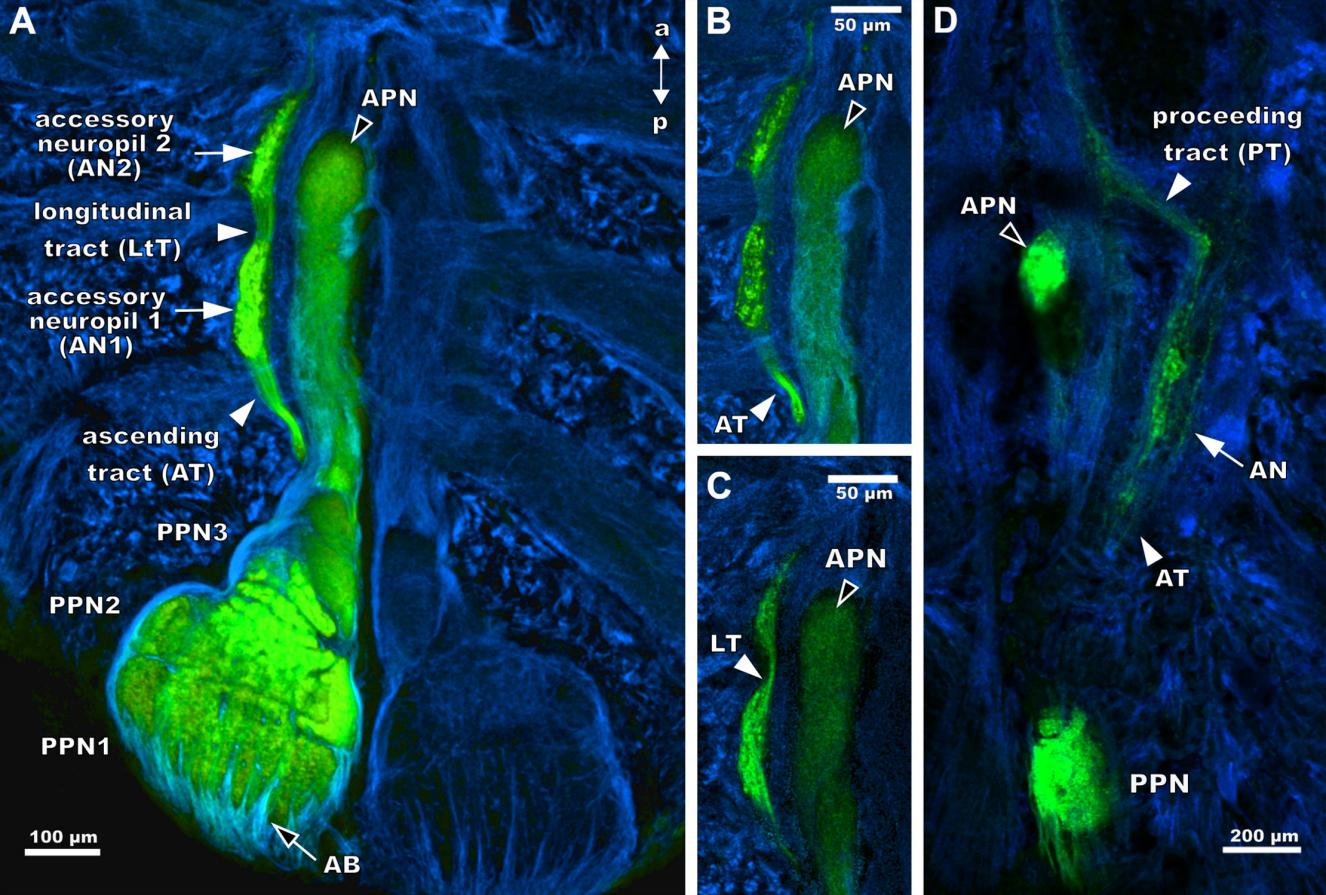
Innervation of the pecten neuropils by sensory axons in *Mesobuthus eupeus* and *Heterometrus petersii*. Maximum projections of horizontal cLSM stacks. Tubulin-immunoreactivity (blue) and anterograde tracing of afferents (green). (A) The entire pecten neuropil exhibits innervation by pecten afferents. Afferent bundles (AB) of the pecten nerve project into the posterior pectines neuropil 1 (PPN1) (white outlined arrow). Adjacent accessory pecten neuropils 1 and 2 (AN1,2) (white arrows), the ascending (AT) and the longitudinal tract (LtT) (white arrowhead) exhibit Neurobiotin labelling. (B) Ventral section of anterior pecten neuropil (APN) and adjacent neuropils. Anterior to PPN3, the ascending tract (arrowhead) proceeds parallel to the APN and innervates the AN1. (C) The longitudinal tract connects the AN1 and AN2 (white arrowhead) dorsally. (D) Anterograde tracing of the entire pecten nerve results in labelling in PPN, APN and AN of *Heterometrus petersii*. Ascending tract emerges anterior to the walking leg neuromere 4 (WLN4) and innervates the accessory neuropil. Proceeding tract leaves the AN anteriorly. Abbreviations: a: anterior; AB: afferent bundles; AN1,2: accessory neuropil 1, 2; APN: anterior pecten neuropil; AT: ascending tract; LtT: longitudinal tract; p: posterior, PPN1-3: posterior pecten neuropil 1–3; PT: proceeding tract; WLN3-4: walking leg neuromere 3–4.

Neurobiotin application to the entire pecten nerve reveals all projection areas of the pectines in the pecten neuropil and adjacent regions. The pecten nerve enters and innervates the PPN from the postero-ventral side. The projections pervade the pecten neuropil in smaller afferent bundles and innervate different areas of PPN1(compare Figs 6 and 7A).

The filled afferent fibres are visible in all posterior pecten neuropil areas (PPN1, PPN2 and PPN3), as well as in the APN (Fig 7A). Furthermore, a sickle-shaped ascending tract of fibres that emerges at the level of the anterior end of PPN3 is labelled in the synganglion (Fig 7A) This tract enters an elongated medial neuropil that is located next to the APN, between the second and the fourth walking leg neuromere (Fig 7A and 7B). This accessory pecten neuropil 1 (AN1) is connected by a longitudinal tract to a similar shaped structure, the AN2 (Fig 7C).

The neuronal tracing of the afferent fibres of the entire pecten nerve of *Heterometrus petersii* exhibits labelling in all parts of PPN and APN and reveals an AN at the level of the fourth walking leg neuromere (Fig 7D). The AN receives input from an ascending tract that emerges at the level of the fourth walking leg neuromere. After exiting the AN, an anteriorly proceeding tract branches off in direction to the ganglion midline.

### Innervation of the pecten neuropils from individually labelled pegs in *Mesobuthus eupeus*

**Fig 8:**
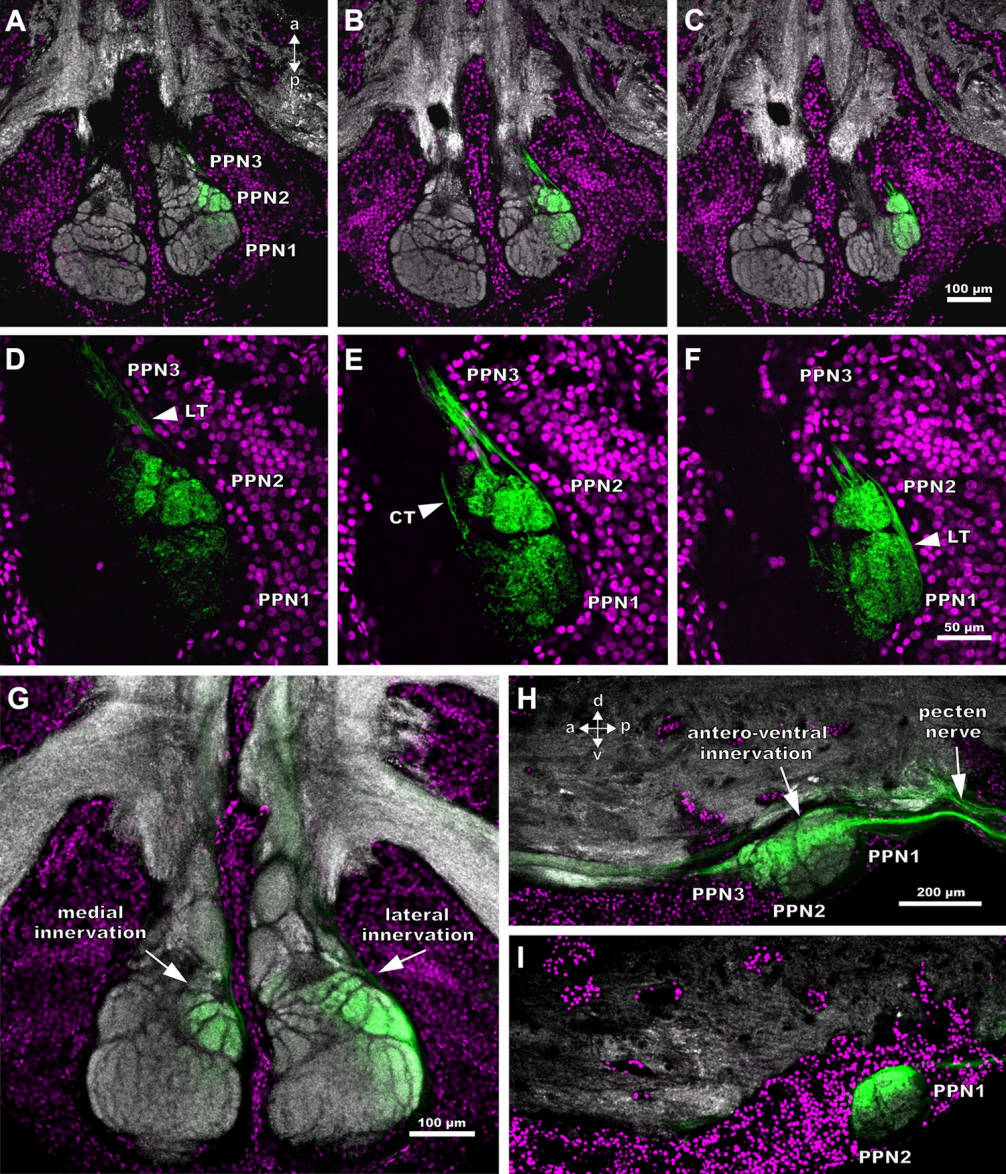
Projection pattern of single pegs within the pecten neuropil of *Mesobuthus eupeus*. Maximum projections of cLSM stacks. Synapsin-immunoreactivity (grey (not shown in D-F)), anterograde tracing (green) and nuclei staining (magenta). (A-C) Horizontal serial sections arranged from ventral (A) to dorsal (C). (D-F) Insets of A, B and C in a higher magnification. (A) I Innervation is restricted mainly to the lateral part of the posterior pecten neuropil 2 (PPN2) ventrally. (B, C) Gradual dorsal innervation of lateral PPN1 areas. (D) Lateral tract (LT) projects anteriorly from the margin of the neuropil (white arrowhead). (E) Small tract (CT) connects PPN1 and PPN2 (white arrowhead). (F) Lateral tract proceeds anteriorly (white arrowhead). (G) Innervation pattern of distal peg afferents in the right pecten neuropil and a proximal peg in the left pecten neuropil (horizontal view). Proximal pegs project medially into the PPN2, whereas distal pegs project laterally towards PPN1 and PPN2. (H) Sagittal view of the posterior part of the synganglion with focus on PPN which protrudes ventrally. The pecten nerve splits into two tracts. The dorsal tract innervates the postero-ventral synganglion and proceeds anteriorly. The ventral tract innervates PPN dorso-laterally and exits the neuropil anteriorly. (I) Sagittal view of a rather lateral section. Innervation of the PPN is restricted to its dorsal part. Abbreviations: a: anterior; CT: connecting tract; d: dorsal; LT: lateral tract; p: posterior, PPN1-3: posterior pecten neuropil 1–3; v: ventral.

The projection areas of single pegs differ, depending on the position of the peg on the pecten. Forwardfills of distally located pegs reveal specific labelling in the lateral parts of the pecten neuropil, while the remaining neuropil areas contain no labelling. These projections enter the posterior pecten neuropil along an afferent tract from the postero-dorsal direction. They proceed laterally and innervate the lateral parts of the PPN1 and PPN2, as well as the outer margin of the PPN3 (Fig 8A–8F). PPN1 and PPN2 are enveloped and interconnected by fibre bundles as well (Fig 8D–8F). The arborisations of this tract spread across and innervate the single lamellae in PPN1 and the glomeruli and the lateral lobules in PPN2. Sagittal sections show that the afferent axons from distal pegs enter the synganglion ventrally from the posterior side via the pecten nerve but are divided into two main tracts (Fig 8H). One tract enters the synganglion ventrally and proceeds anteriorly, while the other tract enters the posterior pecten neuropil dorsally.

Innervations by afferents of a distal peg follow an innervation gradient from dorsal to ventral and lateral to medial, with the strongest labelling in dorso-lateral areas (Fig 8G and 8H). Horizontal sections in more ventrally located parts show prominent labelling of afferents in the lateral lobules and the glomeruli in PPN2 and rather sparse labelling in PPN1 (Fig 8A and 8D). A comparatively uniform innervation of PPN1 and PPN 2 is indicated dorsally (Fig 8B–8F).

The axonal tract converges anterior to the PPN and proceeds from the lateral margin of the pecten neuropil to terminate in the APN. The innervation pattern within the APN appears homogenous and not restricted to a specific area.

Afferents of proximal pegs innervate neuropil areas near the ganglion midline (Fig 8G). The afferent fibres enter the PPN medially from the posterior end via the pecten nerve. A tract that runs along the inner margin of the posterior pecten neuropil innervates PPN2 dorsally (Fig 8G). The intensity of tracer labelling in both PPN differs noticeably in horizontal sections. Projections of proximal pegs concentrate in more ventro-medial regions of the pecten neuropil, compared to distal projections (Fig 8G). After exiting the PPN, the afferents innervate the APN.

**Fig 9:**
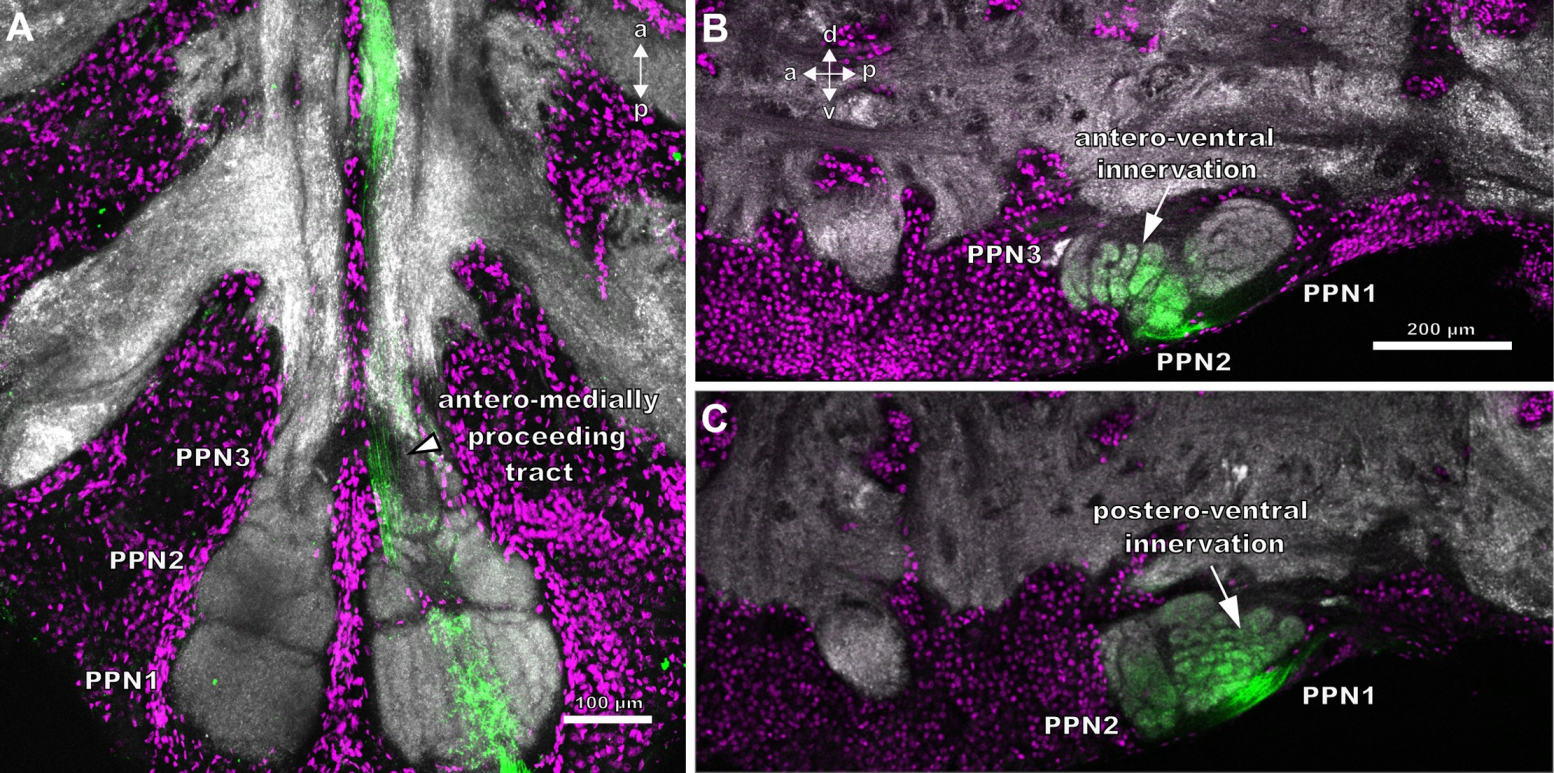
Median pegs innervate central areas within the pecten neuropil of *Mesobuthus eupeus*. Maximum projections of cLSM stacks. Synapsin-immunoreactivity (grey), anterograde tracing (green) and nuclei staining (magenta). (A) Medially situated pegs innervate median parts of the posterior pecten neuropils 1 and 2 (PPN1, 2) (horizontal view). The anteriorly proceeding tract is located near the ganglion midline (arrowhead) and terminates in the anterior pecten neuropil (APN). (B) PPN2 and PPN3 are innervated antero-ventrally by medial peg afferents (white arrow) (sagittal view). (C) In a more lateral section, the innervation is postero-ventral in PPN1 (white arrow). Abbreviations: a: anterior; d: dorsal; p: posterior; PPN1-3: posterior pecten neuropil 1–3; v: ventral.

Afferents of median pegs enter PPN1 postero-ventrally via the pecten nerve (Fig 9C) and innervate PPN2 antero-ventrally (Fig 9B). The peg afferents project to the central parts of PPN1 and PPN2 but innervate PPN3 medially, near the ganglion midline (Fig 9A). The innervation area is restricted mainly to the lamellae of PPN1 and to the glomeruli of PPN2. Most of the innervation of median pegs is concentrated in ventro-medial planes (Fig 9B and 9C). The entire APN is labelled but labelling intensity is more pronounced on the ventral side.

### Projections of single pegs in the pecten neuropil of *Heterometrus petersii*

**Fig 10:**
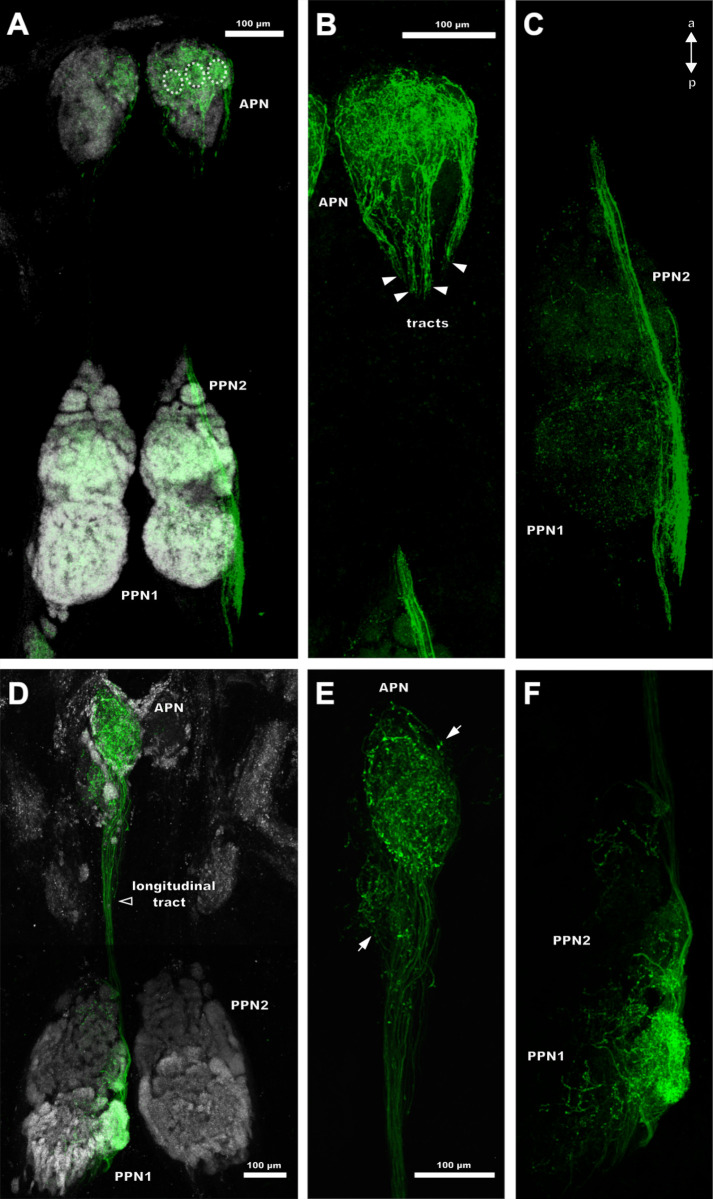
Innervation of the posterior pecten neuropil (PPN) of *Heterometrus*. *petersii* by distal (A-C) and proximal (D-F) peg afferents. Maximum projections of horizontal cLSM stacks. Synapsin-immunoreactivity (grey (not shown in B, C and E, F)) and anterograde tracing (green). (A) Tract from the pecten nerve enters the synganglion from posterior and proceeds laterally along the margin of the posterior pecten neuropil (PPN). PPN1 and 2 are innervated laterally. Furthermore, the entire anterior area of the anterior pecten neuropil (APN) is innervated by afferents. The innervation pattern reveals conspicuous glomerular-like structures (dotted outlines). (B) Higher magnification of APN from (A). Longitudinal tract splits into distinct branches (white arrowheads), each innervating a glomerular-like structure of APN. (C) Higher magnification of the PPN from (A). The labelled tract is thicker at the posterior end and finer at the anterior end. (D) Projection pattern of proximal peg afferents within the pecten neuropil. Longitudinal tract connects PPN and APN, the former being medially innervated by afferent fibres. (E) Higher magnification of APN from (D). The longitudinal axons terminate in two ellipsoid subcompartments in APN. (F) Higher magnification of the PPN from (D). The main projection area is located laterally and branches into the PPN1 and 2. Abbreviations: a: anterior; APN: anterior pecten neuropil; PPN1,2: posterior pecten neuropil 1, 2; p: posterior.

The innervation patterns associated with single pegs in varying positions along the pectines appears to be very similar to those in *M*. *eupeus*. The pecten nerve enters the pecten neuropil postero-ventrally and splits into afferent bundles at the posterior end of PPN1 (Fig 10). Afferent projections from distal pegs innervate lateral parts of the neuropil, whereas afferents from proximal pegs project in medial areas (Fig 10A and 10D). In contrast to *M*. *eupeus*, innervation by afferents from single pegs is restricted to smaller areas within the neuropil (Fig 10C and 10F). Similar to the projections in *M*. *eupeus*, single pegs innervate subcompartments of PPN (Fig 10C and 10F). Axon bundles leave the posterior neuropil via a longitudinal tract that is located near the ganglion midline and split into finer bundles before entering the APN (Fig 10B). In case of distal peg afferents, four tracts enter the APN and innervate at least three bulbous subregions (Fig 10B). Yet, present results do not allow a clear judgement whether or not the number of these spheroid compartments correspond to that of the tracts. Proximal peg afferents project to two ellipsoid subregions of the APN (Fig 10E). One of these subregions is smaller and located anterior to the fourth walking leg neuromere, the other one is larger and terminates at the level of the third walking leg neuromere. Nonetheless, the afferents of proximally and distally located pegs innervate the APN of *H*. *petersii* uniformly (Fig 10B and 10E).

### Differential anterograde tracing of adjacent pegs

**Fig 11:**
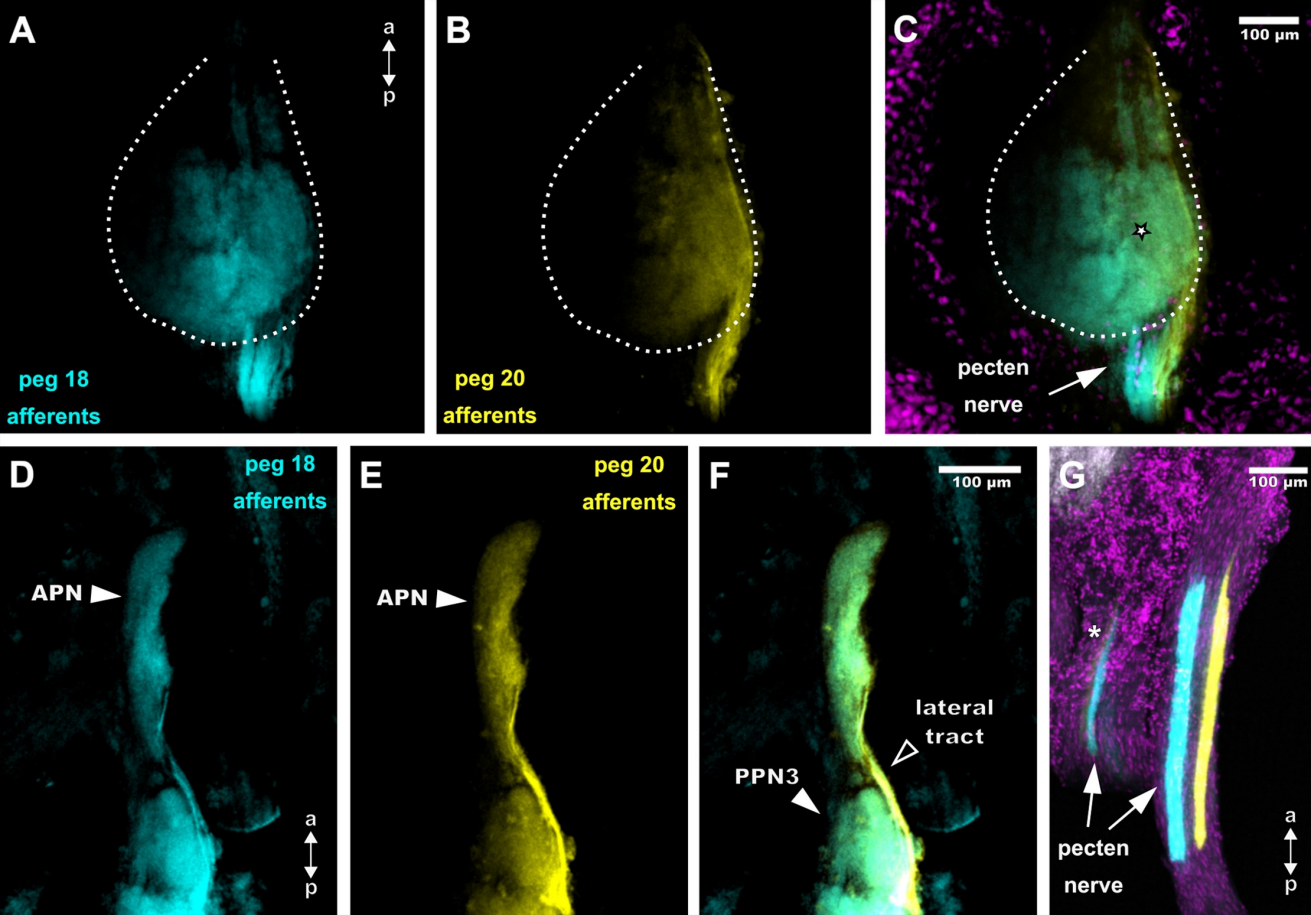
Double-labelling of two adjacent distal pegs in *Mesobuthus eupeus* with lipophilic markers. Maximum projections of horizontal cLSM stacks. Nuclear labelling in magenta. DiA (yellow) was used for peg 20 (second to last distal peg), DiI (cyan) for peg 18 (fourth to last distal peg). (A) The innervation of peg 18 is distributed medially (dotted line, neuropil outline). (B) Afferents of peg 20 stay on the very lateral side of the posterior pecten neuropil (PPN) and the labelled tract runs along the lateral neuropil margin (dotted line). (C) Merge of (A) and (B). The afferent projections hold a specific spatial position within the pecten nerve but partially overlap in the neuropil (outlined star) (dotted line, neuropil outline). (D) Innervation of the entire anterior pecten neuropil (APN) by afferents of peg 18 (cyan). (E) Innervation of the entire APN by afferents of peg 20 (yellow). (F) Merged image of (D) and (E). White arrows indicate PPN3 and a lateral tract. The afferents of both pegs innervate APN in a similarly homogeneous fashion. (G) Pecten nerve with afferent fibres of peg 18 (cyan) and 20 (yellow) indicated by arrows. Afferents of peg 20 (cyan) of the left pecten indicated by asterisk. Abbreviations: a: anterior; APN: anterior pecten neuropil; PPN3: posterior pecten neuropil 3; p: posterior.

To determine how adjacent peg afferents innervate the posterior pecten neuropil, several animals of *M*. *eupeus* were treated for differential afferent tracing (Fig 11). Two distal pegs (peg 18 and peg 20) were treated with lipophilic dyes and their projections to the pecten neuropil were examined. The pecten nerve contains two distinctly labelled afferent fibre bundles when entering the synganglion corresponding to the two labelled pegs (Fig 11G). The afferents associated with peg 20 run more laterally within the pecten nerve, whereas the axon bundle of peg 18 is located medially. The fibres between the two labelled bundles are devoid of any signal. After entering the PPN, the projection areas of the two labelled pegs are partially superimposed, but each peg projection exhibits a specific innervation area (Fig 11A–11C). Afferents of pegs 18 and 20 innervate lateral parts of the pecten neuropil, but afferents of peg 20 supplies areas closer to the lateral margin of the neuropil, whereas peg 18 afferents project to areas slightly closer to the centre of the neuropil (see Fig 11A and 11B). There is an overlap among the main innervation areas of the two pegs (Fig 11C). After exiting the PPN in anterior direction, both afferent axon bundles merge laterally at the level of PPN3 and innervate the APN evenly. Innervation of the APN by individual peg afferents is not restricted to a distinct region and appears to be rather homogenous (Fig 11D–11F).

## Discussion

Concerning the chemosensory pathway of scorpions, previous studies have addressed the external and internal morphology of the pectines [11,75,77,78,103], their mechano- and chemosensory functions [81,86,88,91,92,104,105] sensilla response properties [104,106,107], and neuronal connectivity and processing [11,77,94,108,109]. Our comparative analysis highlights differences and similarities in the morphology and innervation of the pecten neuropils in two different scorpion species. The goal is a better understanding of the mode of processing of sensory inputs within the primary chemosensory pathway of scorpions.

### Comparison of the pecten neuropils in scorpions

**Fig 12:**
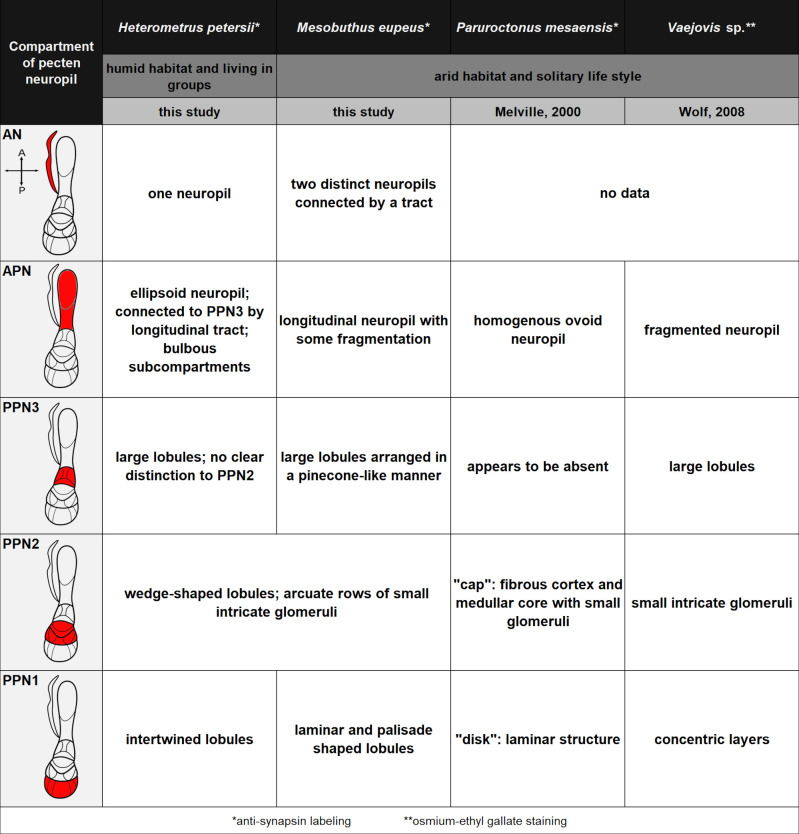
Overview of variations in the anatomy of the pecten neuropils in *Mesobuthus eupeus*, *Heterometrus petersii*, *Paruroctonus mesaensis* and *Vaejovis* sp. Habitats and life styles of the respective scorpion species are listed under the species names. Left column depicts a generelised scheme of the pecten neuropil; relevant subregions are named in the upper left corner and marked in red. Species-specific differences of each subregion are listed under the respective scorpion species. Results in *Paruroctonus mesaensis* and *Vaejovis* sp. were taken from neuroanatomical studies of J.M. Melville [74] and H. Wolf [11], respectively. Asterisks refer to the used staining method. Abbreviations: a: anterior; AN: accessory neuropil; APN: anterior pecten neuropil; PPN1-3: posterior pecten neuropil1-3; p: posterior.

**Fig 13:**
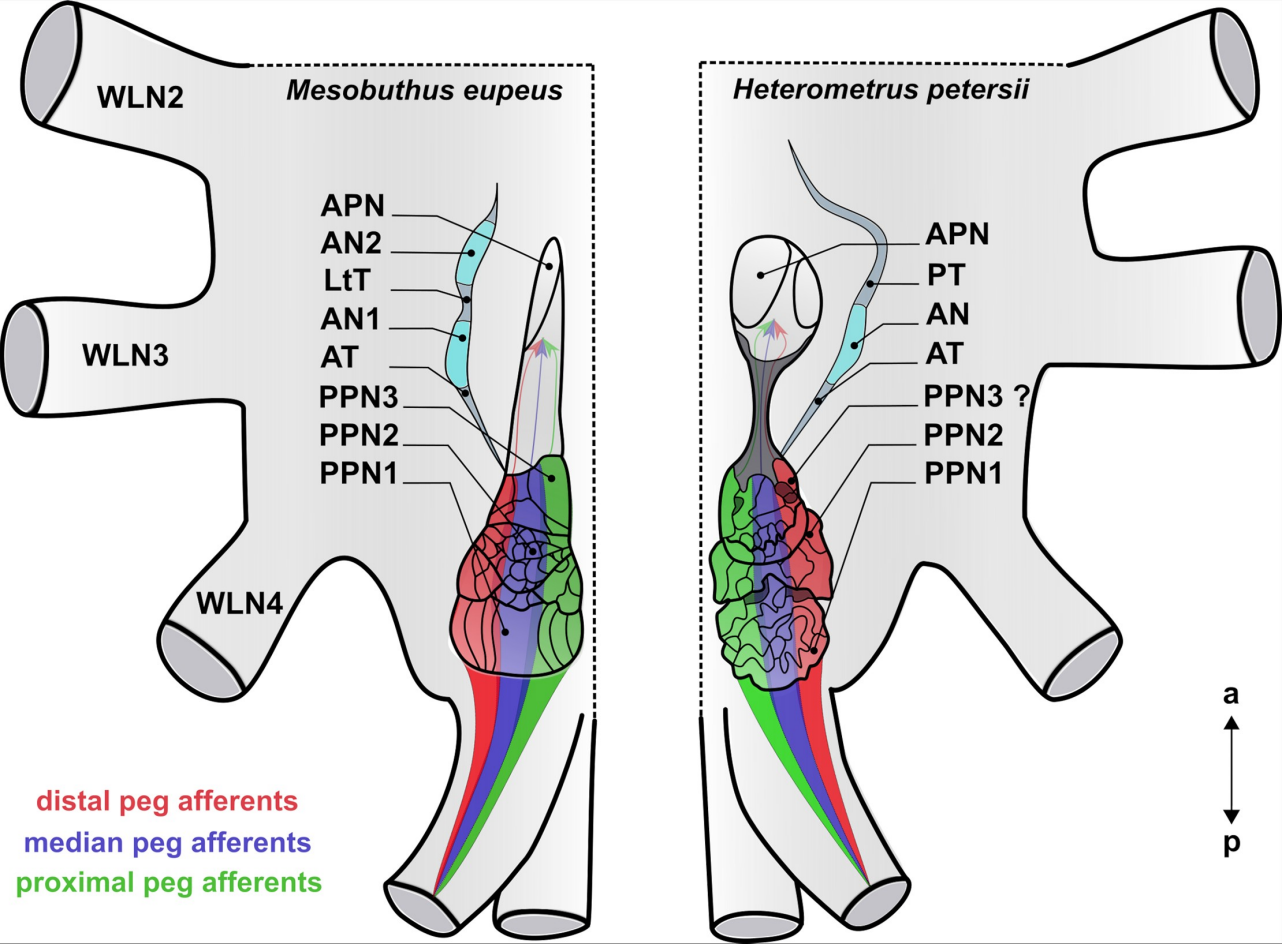
Schematic summary of the pecten neuropils and their innervation in *Mesobuthus eupeus* (left) and *Heterometrus petersii* (right). Posterior part of the synganglion in horizontal section. The pecten neuropil of *M*. *eupeus* exhibits a compartmentalisation into different subregions: The anterior pecten neuropil (APN) at the level of walking leg neuropil 3 (WLN3), the accessory neuropils 1, 2 (AN1,2) (cyan) and the posterior pecten neuropils1-3 (PPN1-3) in the more posterior part of the synganglion. PPN consists of three conspicuous subregions: The laminar PPN1, the glomerular PPN2 and the lobular PPN3. The pecten neuropil of *H*. *petersii* contains an ellipsoid APN, an AN (cyan) and a PPN with probably two main regions (PPN1 and 2). The structures of PPN1 and PPN2 are intertwined, although PPN2 appears more lobular. The PPN of *H*. *petersii* is interspersed with somata. The subregions of the PPN are delimited (indicated by dark grey areas in PPN1 and PPN2). PPN and APN are connected by a longitudinal tract (indicated by dark grey area between PPN and APN. The innervation of peg afferents exhibits a somatotopic organisation in the PPN: afferents of distal pegs project into lateral regions (red), afferents of median pegs into the central region (blue) and proximal peg afferents into medial regions (green) of the PPN. Innervation areas overlap, but the exact quantity of afferent overlap is not known. APN is innervated homogenously by all afferent fibres. Abbreviations: a: anterior; AN1,2: accessory neuropil1, 2; APN: anterior pecten neuropil; AT: ascending tract; LtT: longitudinal tract; p: posterior; PPN1-3: posterior pecten neuropil 1–3; PT: proceeding tract; WLN2-4: walking leg neuromere 2–4.

We analysed the morphology of the pecten neuropils of the two scorpion species *Mesobuthus eupeus* (Koch, 1839) and *Heterometrus petersii* (Thorell, 1876), two species living in different habitats and being phylogenetically rather distantly related [110]. Besides the two representatives of the families Buthidae and Scorpionidae investigated in this account, several aspects of the pecten neuropils are known from the family Vaejovidae (*Vaejovis spinigerus* and *V*. *flavus* [11], and *Paruroctonus mesaensis* [74]). These previous studies examined the afferent innervation of pecten neuropils in desert-dwelling scorpions by tactile hairs [74] as well as general morphology and innervation by chemosensory afferents [11]. Regardless of the obvious ecological and phylogenetical differences, our results on the morphology of the pecten pathway are rather similar for the two examined species. This suggests a comparable pecten neuropil organisation, which has been shown to be true to some extent (Figs 4, 5, 12 and 13). This includes (i) the presence of two major subregions in the pecten pathway, posterior pecten neuropil (PPN) and anterior pecten neuropil (APN), (ii) a subdivision of the posterior pecten neuropil into at least two areas (PPN1 and 2), (iii) the presence of at least one accessory neuropil, (iv) a somatotopic organisation of the peg afferents along a medio-lateral axis in the PPN, (v) and a lack of somatotopy in the APN. The most striking differences include the internal compartmentalisation and the organisation of the subcompartments of the pecten neuropils that appear to be taxon-specific (summarised in Figs 12 and 13).

While the structure of PPN1 ranges from palisade-shaped lobules (*M*. *eupeus*), to concentric layers (*Vaejovis* sp. [11]), the structure of PPN2 appears rather consistent across different scorpion species. This consistency most notably includes the presence of small intricate glomeruli (Fig 12). Glomeruli normally refer to chemosensory processing units in a neuroanatomical context [3], which might suggest a similar situation in the pecten neuropils. Due to the bimodal nature of the peg sensillae, however, it is not clear whether these glomeruli receive input only from chemosensory axons or whether the mechanosensory afferents also project into these structures.

### Anterior extension of the pecten neuropil

The APN is an anterior extension of the pecten neuropil that displays a variety of different shapes, structures and sizes (Figs 12 and 13). While in *H*. *petersii* it is ovoid, large and consists of spherical subcompartments, the APN of *M*. *eupeus* is strongly elongated and only subtly fragmented (Fig 4A and 4D).

Interestingly, an anterior extension of the first integration centre of chemosensory afferents is known from Solifugae [12,111,112]. The solfugid chemosensory neuropil, consisting of several glomeruli, is positioned ventro-medially, in an anterior position near the pedipalpal neuromeres, although the primary chemosensory organs–the malleoli–are positioned on the fourth walking legs [12,64,71,113]. This anterior relocation of chemosensory neuropils in both taxa might be beneficial in the further signal processing (e.g. relaying pre-processed stimuli to higher integration centres) and integrating input from anterior sensory structures. The pedipalps of scorpions and solifuges bear special sensory organs (e.g. [54,89,114–116]), including chemosensory fields (e.g. the constellation array in scorpions [115,116]). Whether or not the chemosensory sensillae of the pedipalps send their axons into the APN or malleolar neuropil, respectively, remains completely open. However, a close apposition of the two initial (chemo-)sensory integration centres might facilitate common processing of stimuli from pedipalps and pectines/malleoli before transmission to higher-order integration centres.

If future studies confirmed the latter hypothesis, the internal arrangement of the APN might be related to the complexity of the constellation array. In *M*. *eupeus*, where the APN is rather small and homogenously structured, the constellation array consists of only five chemosensory sensillae [115]. The number of sensillae in constellation arrays of *V*. *spinigerus*, *V*. *flavus* and *P*. *mesaensis* has not been investigated, but other species in these genera have only four to five and two sensillae, respectively. Unfortunately, no information concerning the constellation array of *H*. *petersii* is available, but an examination in this context is crucial to evaluate this hypothesis.

### Structural variation potentially associated with habitat or sociality

Scorpions from both humid and arid habitats exhibit similarities in significant details, concerning basic anatomy and structure in their chemo- and mechanosensory pathways (see above). Major differences were observed in the inner neuropil structures of the respective subregions and the sizes and structures of the APN (Figs 12 and 13). When speculating from the two species studied here, scorpions living in humid ecological niches may exhibit more intricate and intertwined neuropilar structures in their PPN, while scorpions from arid biotopes may have more homogeneously structured pecten neuropils (Figs 12 and 13). Furthermore, the APN in tropical species may be more ovoid, have glomerular parcels and may connect to the PPN by a longitudinal tract, while the APN in desert scorpions may be elongated and cone-shaped.

Interestingly, numbers of pegs as well as peg sensillae per peg seem to be related to environmental conditions. In general, desert-dwelling species possess a larger number of pegs, compared to species inhabiting humid regions [11,77]. A reasonable explanation might be the rather long stability of chemosensory cues in humid surroundings, compared to arid areas [117]. In addition, the social structure might be of importance as well. Desert scorpions like *M*. *eupeus* are often solitary, probably by necessity when considering low prey densities and relatively high predator pressure [118], while species from tropical forests like *H*. *petersii* often live in groups [86,119]. While the numbers of peg sensillae are smaller in *H*. *petersii* compared to most desert scorpions, nothing is known about receptor complexity in any scorpion species. Maybe the diversity of receptors encoding different chemosensory cues is relatively high in scorpions inhabiting humid environments and/or with social lifestyle–resulting in a comparatively more convoluted and intricate pecten neuropil (Figs 12 and 13). More solitary desert scorpions might have lower receptor diversity and less complex neuropils. The latter may provide an advantage with regard to the presentation of higher receptor numbers and higher sensitivity that would appear necessary in arid environments [117]. Thus, the high number of sensillae in the latter might not reflect a putatively low resolution for different odours but a requirement for high sensitivity to detect more transient cues and find prey and mates. Alternatively, all of the above features may reflect a phylogenetic signal.

### Projection pathways of unimodal mechanosensory hairs associated with the pectines

Besides the bimodal peg sensillae, the pectines are equipped with unimodal mechanosensory hair sensillae, mainly distributed on lamellae and fulcra (Fig 2). While we have only traced the peg sensillae in most of our experiments, we carried out forward fills of the entire pecten nerve, which houses the afferents of the bimodal peg sensillae, as well as the mechanosensory hair sensillae. Only in these entire nerve tracing experiments, we observed an additional, hitherto undescribed neuropil in the central nervous system (CNS), which is positioned laterally to the anterior extension of the pecten neuropil (associated pecten neuropil, see Fig 7). This leads to the preliminary conclusion that afferents of the unimodal mechanosensory hair sensillae project towards these accessory pecten neuropils and thus are processed separately from the bimodal peg sensillae. In contrast to our observations, Melville [74] showed that mechanosensory hair sensillae on the pecten marginal lamella of *Paruroctonus mesaensis* project to the cortex of the cap-like pecten neuropil portion in a somatotopic fashion. The cortex of the cap-like neuropil part in Melville’s study appears to correspond to the bilateral lobules of PPN2 in the present investigation. According to Melville’s observation, it appears possible that the scorpion pectines possess different populations of unimodal mechanosensory hair sensillae with divergent projection patterns. Thus, Melville [74] labelled a population of sensillae that project towards the PPN2 cortex, while other populations might show separate more anteriorly located neuropil targets, namely in the accessory pecten neuropils that run laterally to the anterior pecten neuropil. This might further imply that the PPN2 receives input from both bimodal and unimodal mechanosensory neurons. It was not within scope of this paper to address the projection patterns of the mechanosensory hair sensillae, while drawn assumptions are relevant for the following comparison of uni- and bimodal chemo-/mechanosensory systems within Arthropoda.

### Comparison of chemosensory pathways: Unimodal versus bimodal systems—chemotopy versus somatotopy

Our knowledge of chelicerate sensory processing is rather limited when compared to that of other arthropod lineages, especially insects. While chelicerates do not possess dedicated chemosensory appendages associated with the second head neuromere, they evolved diverse alternatives to cope with the lack of antennal structures. The sensory equipment and thus the functionality of these intriguing structures ranges from airborne olfaction in Acari [50,67–70] and harvestmen [120,121] through unimodal contact chemosensation in solifuges [12,64] to bimodal chemo-/mechanosensation in scorpions [11,73,74,77,94]. However, central projections of sensory afferents have been traced only in Acari, Araneae and Solifugae [12,35,69,122]. In Solifugae, the malleoli appear to bear only unimodal contact-chemosensory sensillae with a supposed chemotopic representation in the malleolar neuropil with glomerular organisation [12]. The same holds true for the olfactory lobes in mites and ticks [78,122]. Thus, unimodal chemosensory systems, be it airborne or contact chemosensation, show high correspondences in the anatomy of their first integration centre to Mandibulata (compare arachnid unimodal systems described above to e.g. the olfactory system of mandibulate antennae: [7,13,14,15–21,123–125]). These similarities in unimodal olfactory systems have also been noted from other invertebrates and vertebrates [3,20,21,125].

Spiders possess bimodal contact chemoreceptors on their legs. This system has been studied in considerable detail in the wandering spider *Cupiennius salei*. Here, axons of bimodal hair sensillae on the legs project somatotopically into central longitudinal neuropils [22]. These are subdivided into subcompartments processing different types of sensilla input, originating from different regions on the cuticle ([22], see their Fig 11). Thus, the central projections from both modalities (chemosensation as well as mechanosensation) innervate the same structures. No glomerular substructures have been identified in the target regions of the bimodal leg sensillae. The lack of identifiable glomerular structures might be due to the comparably low number of contact chemosensory sensillae on the distal parts of the appendages (200 on the pedipalps in *C*. *salei* [126], in comparison to several thousand on scorpion pegs [11]).

The bimodal projection pattern has correspondences not only in scorpions and spiders. In locusts, for example, both chemo- and mechanosensory axons associated with bimodal sensillae project into the same region of the respective neuropil. The mechanosensory axons are organised in a somatotopic fashion, and the chemosensory projections follow a similar organisation. This demonstrates that chemosensory input can be represented in a somatotopic manner, where applicable together with the respective mechanosensory input. Again, glomerular organization of the chemosensory aspect might not be detectable due to comparably low numbers of chemosensory afferents. In Crustacea the antennular bimodal sensillae project to the paired lateral antennular neuropils, where both modalities appear to have a somatotopic organization (e.g., [29]). However, other studies revealed a clear separation of the two modalities projecting to distinct neuropils [127]. Future investigations on other representatives of arachnids as well as mandibulates, are crucial to shed light on the neuroanatomical characteristics and the evolution of bimodal pathways.

## Conclusions and outlook

The scorpion primary chemosensory pathway displays several idiosyncratic features: (i) compartmentalisation into a tripartite posterior pecten neuropil (PPN1-3) and one anterior pecten neuropil (APN), with each respective subregion having a distinct neuropil structure, presumably processing different sensory aspects; (ii) parallel innervation of all four neuropil regions, (iii) somatotopic organisation of bimodal peg afferents along a medio-lateral axis, resulting in a first integration centre for spatial qualities of chemosensory input and high resolution of substrate-borne chemical gradients.

Our experiments cannot distinguish between the mechanosensory and chemosensory projection patterns of the peg sensillae, however, our data allow speculations about the spatial distribution of these modalities. It appears evident that every peg occupies its own projection area within the PPN (even though overlapping to an extent with adjacent peg projections), whereas the APN is always innervated rather homogenously even in single peg forwardfills. This indicates an alignment of more or less identical chemosensory organs–the pegs–along the somatotopic innervation of the PPN, conserving positional information for both mechanosensory and chemosensory inputs (Fig 13). This idea is supported by the observation that each peg is equipped with a similar set of peg sensillae concerning their chemosensitivity [90]. At the same time, a chemotopic representation of the chemosensory afferents might be present in the glomerular or lobular portion of an individual peg’s projection area. The APN might be a first integration centre for peg inputs in the PPN where local interneurons as well as projection neurons (and potentially input from the pedipalpal sensory system, see above) converge to generate output to higher integration centres. These latter assumptions are speculative, of course, and require further study, preferentially by labelling particular subsets of chemosensory receptor neurons. Besides function and connectivity of the APN, our next steps will focus on the projections to higher integration centres in order to understand how bimodal information is processed and how it relates to different behaviors.
